# Membrane-Mediated Interactions Between Nonspherical Elastic Particles

**DOI:** 10.1021/acsnano.2c05801

**Published:** 2023-01-20

**Authors:** Jiarul Midya, Thorsten Auth, Gerhard Gompper

**Affiliations:** Theoretical Physics of Living Matter, Institute for Biological Information Processing and Institute for Advanced Simulation, Forschungszentrum Jülich, 52425 Jülich, Germany

**Keywords:** cellular particle uptake, passive endocytosis, soft particles, vesicles, wrapping, membrane-mediated interactions, continuum membrane model

## Abstract

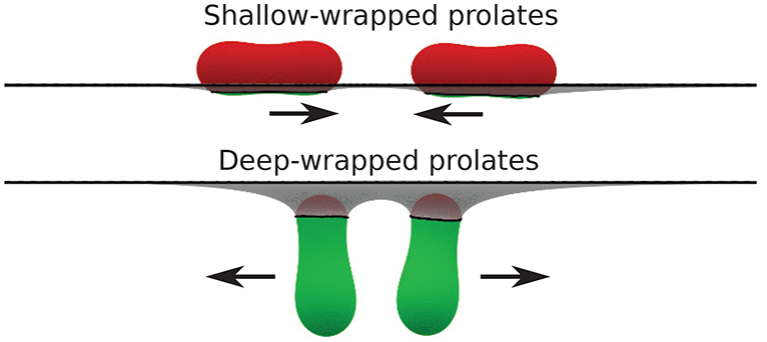

The transport of particles across lipid-bilayer membranes is important for biological cells to exchange information and material with their environment. Large particles often get wrapped by membranes, a process which has been intensively investigated in the case of hard particles. However, many particles in vivo and in vitro are deformable, e.g., vesicles, filamentous viruses, macromolecular condensates, polymer-grafted nanoparticles, and microgels. Vesicles may serve as a generic model system for deformable particles. Here, we study nonspherical vesicles with various sizes, shapes, and elastic properties at initially planar lipid-bilayer membranes. Using the Helfrich Hamiltonian, triangulated membranes, and energy minimization, we predict the interplay of vesicle shapes and wrapping states. Increasing particle softness enhances the stability of shallow-wrapped and deep-wrapped states over nonwrapped and complete-wrapped states. The free membrane mediates an interaction between partial-wrapped vesicles. For the pair interaction between deep-wrapped vesicles, we predict repulsion. For shallow-wrapped vesicles, we predict attraction for tip-to-tip orientation and repulsion for side-by-side orientation. Our predictions may guide the design and fabrication of deformable particles for efficient use in medical applications, such as targeted drug delivery.

Budding enables biological cells to exchange material with their environment across the plasma membrane.^1^ In the context of uptake of single particles, budding is often, more specifically, referred to as wrapping. Wrapping processes of mesoscopic particles by cell membranes are abundant in nature, such as entry and exit of viruses and parasites into host cells.^2,3^ Furthermore, extracellular vesicles can be taken up via endocytosis.^4^ Therefore, wrapping of particles at lipid-bilayer membranes is also important for the design of diagnostic and therapeutic agents. Particles for medical applications include lipid particles for targeted drug delivery^5,6^ and magnetic nanoparticles that serve as heat sources for cancer therapy.^7^ For hard particles, the desired wrapping states can be achieved by controlling particle shape and size, and particle–membrane adhesion strength.^8^ For soft particles, in addition, particle deformability plays an important role in the wrapping process. For example, an increased stability of partial-wrapped states has been reported for initially spherical vesicles with low bending rigidities of their membrane.^9^ Vesicles flatten upon binding to planar substrates;^10^ the increased stability of partial-wrapped states is therefore similar to the increased stability reported for hard oblate ellipsoidal compared with spherical particles.^11^ Furthermore, soft particles can adjust to constraints and react to external stimuli. Prominent examples are filamentous viruses at plasma membranes that bend,^12^ parasites that squeeze through narrow constrictions when invading host cells through the tight junction,^13^ and SARS-CoV-2 virions, which are enveloped by a lipid membrane and assume nonspherical shapes near cell membranes.^14^

Engineered soft particles encompass a wide variety of architectures with tunable elastic properties: microgels,^15^ star polymers,^16^ polymer-grafted nanoparticles,^17^ polymeric shells,^18^ particles made from dendrimers as building blocks,^19^ vesicles,^20^ and biomolecular condensates.^21^ For example, the elastic properties of microgels can be controlled by cross-linker density and electric charge,^22,23^ of star polymers by functionality and chain length,^24^ of polymer-grafted nanoparticles by grafting density and chain length,^17^ and of unilamellar fluid vesicles by membrane bending rigidity and osmotic concentrations.^25^ Vesicles, in particular, are a versatile and well-established biomimetic system and have also been used as a generic model system for soft particles.^9,26,27^ A zoo of vesicle shapes can be obtained by changing the membrane curvature-elastic parameters and osmotic concentrations, which includes spherical, prolate, oblate, stomatocyte, pear-shaped, and starfish shapes.^20^

Particles have been shown to laterally move on lipid membranes in experiments using viruses on plasma membranes and synthetic particles on free-standing lipid bilayers, as well as in computer simulations.^28−30^ Therefore, long-ranged membrane-mediated interactions can drive self-assembly of partial-wrapped particles at lipid-bilayer membranes.^31,32^ However, the nature of the membrane-mediated interactions can either be attractive or repulsive, depends on many parameters, and is thus not easy to predict. For example, for hard particles, the deformation energy of the membrane and the particle–membrane adhesion energy depend on the distance between interacting particles.^33^ Experimental and theoretical studies show membrane-mediated attraction and cluster formation of hard spherical particles on vesicles.^34^ Hard spherical particles on giant unilamellar vesicles have been found to attract each other and to induce tube formation.^33,35−38^ In vivo, viruses are shed as multivirion clusters in vesicles.^39,40^ Ellipsoidal microgels on giant unilamellar vesicles have been found to form ordered structures with spacings that suggest membrane-mediated mutual repulsion.^15^

In this work, we use energy minimization to calculate and predict shapes and wrapping states for single, nonspherical vesicles that get wrapped at planar membranes. We find that adhesion to a membrane can change the shape of vesicles from prolate to oblate for partial-wrapped states. Wrapping transitions can be continuous and discontinuous; shape and orientation changes of vesicles are always discontinuous with an energy barrier. Increased vesicle softness, decreased reduced vesicle volume, and increased tension of the planar membrane enhance the stability of the partial-wrapped states. We also calculate the membrane-mediated pair interaction between two prolate vesicles in shallow and deep-wrapped states. We predict attraction between shallow-wrapped vesicles in tip-to-tip orientation and repulsion between deep-wrapped vesicles as well as between shallow-wrapped vesicles in side-by-side orientation. For shallow-wrapped vesicles, increasing particle deformability induces a shape change from prolate to oblate and an interaction change from attractive in tip-to-tip orientation to repulsive for oblate vesicles.

## Results and Discussion

### Wrapping Energies

Our predictions for wrapping vesicles at membranes are based on a continuum membrane model where the membranes are represented by mathematical surfaces, which is applicable for particles with radii *r*_p_ ≳ 20 nm. The total energy of the system is calculated using the energy functional
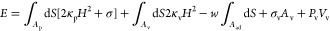
1where *H* = (*c*_1_ + *c*_2_)/2 is the mean curvature and *c*_1_ and *c*_2_ are the two principal curvatures. *A*_v_ is the area of the vesicle membrane. *A*_p_ is the area of the initially planar membrane, and *A*_ad_ is the adhered area of the vesicle membrane. The bending rigidities are κ_p_ and κ_v_ for the planar and the vesicle membrane, respectively; σ is the tension of the planar membrane, and *w* is the adhesion strength between vesicle and membrane. Throughout our study, we assume that the Gaussian saddle-splay modulus vanishes, . The last two terms ensure the conservation of the vesicle surface area *A*_v_ and volume *V*_v_, where σ_v_ and *P*_v_ are the corresponding Lagrange multipliers. The shape of the vesicle is characterized by its reduced volume *v* = *V*_v_/*V*_sph_, where *V*_v_ is the actual volume of the vesicle and *V*_sph_ is the volume of a spherical vesicle with the same membrane area. The relative deformability of the vesicle membrane is characterized by the dimensionless ratio κ_v_/κ_p_. The wrapping fraction *f*_w_ = *A*_ad_/*A*_v_ measures the fraction of the vesicle membrane that is adhered. For convenience, we will use the reduced membrane tension  and the reduced adhesion strength  in the following.

### Wrapping and Shape Transitions for a Nonspherical Vesicle

**Figure 1:**
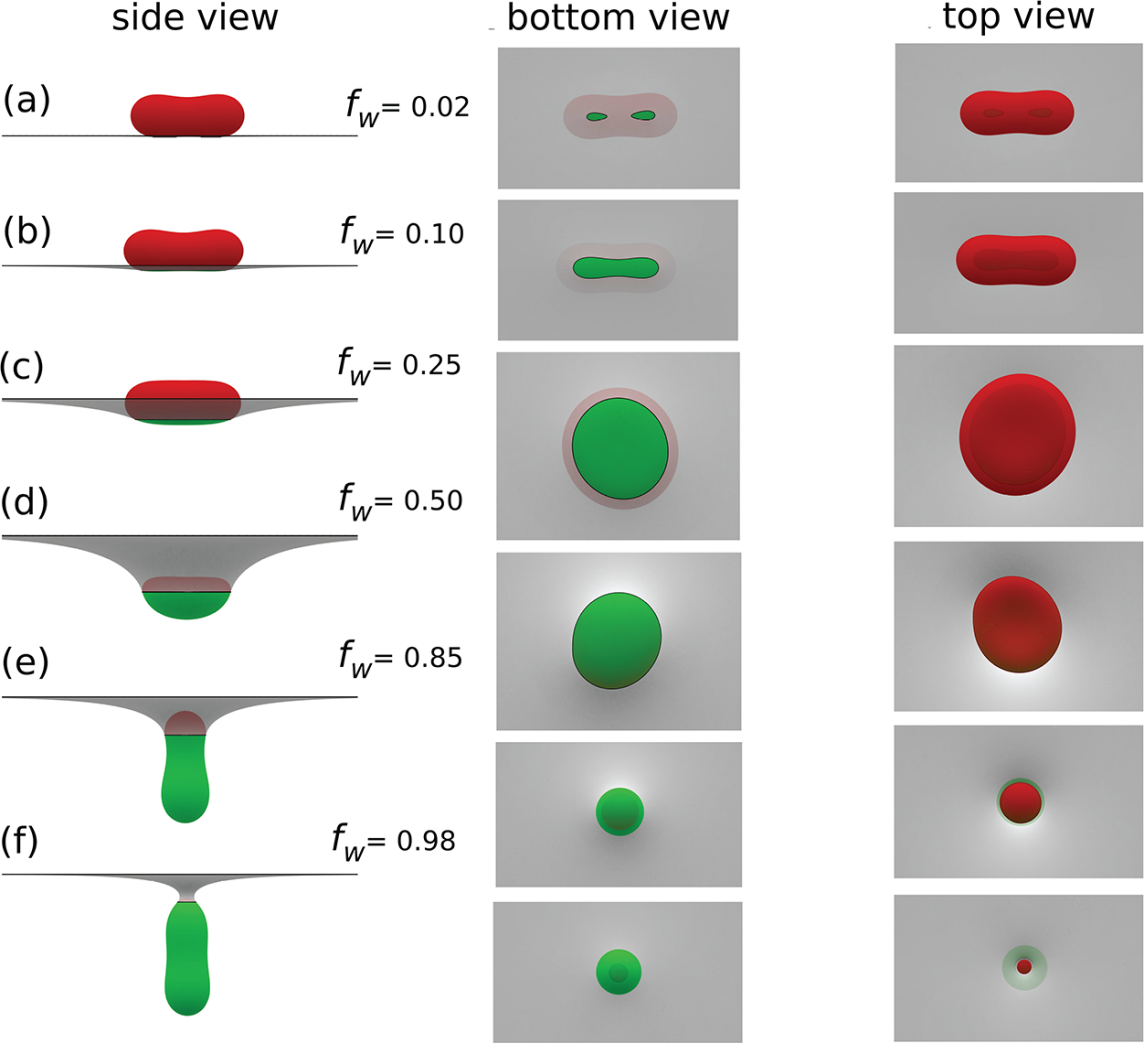
Wrapping of a nonspherical vesicle with *v* = 0.8 at an initially planar membrane for the wrapping fractions (a) *f*_w_ = 0.02 (unstable state), (b) *f*_w_ = 0.10 (stable state), (c) *f*_w_ = 0.25 (stable state), (d) *f*_w_ = 0.50 (unstable state), (e) *f*_w_ = 0.85 (stable state), and (f) *f*_w_ = 0.98 (stable state). The minimum-energy shape of the free vesicle is prolate. The bending rigidity ratio is κ_v_/κ_p_ = 1, and the reduced membrane tension is . The left, middle, and right columns represent the side, bottom, and top views of the vesicle-membrane system, respectively. Snapshots for wrapping of a nonspherical vesicle with higher reduced volume (*v* = 0.95) are presented in Figure S2.

The wrapping of nonspherical vesicles at planar membranes is studied by systematically varying the bending rigidity ratio κ_v_/κ_p_, the reduced volume *v* of the vesicle, and the tension σ of the planar membrane. Figure 1 shows minimum energy shapes of a vesicle with reduced volume *v* = 0.8 at a planar membrane as a function of the wrapping fraction *f*_w_ = *A*_ad_/*A*_v_. Wrapping always starts from the lowest mean curvature region of the vesicle surface; for a prolate vesicle, binding occurs in submarine orientation with its major axis parallel to the membrane (see Figure 1a). As wrapping progresses, the deformation of the vesicle and surrounding membrane increases; see Figure 1b–d. A stable prolate state of the vesicle in submarine orientation is found in the range of wrapping fraction 0.07 ≲ *f*_w_ ≲ 0.11. A further increase in wrapping fraction leads to a shape change of the vesicle from prolate to oblate, and a stable oblate state is found in the range of wrapping fraction 0.18 ≲ *f*_w_ ≲ 0.31. The deformation of the vesicle reaches its maximum at *f*_w_ ≈ 0.5, where the vesicle shape is oblate and the membrane touches the rim of the vesicle where the mean curvature is maximal. For *f*_w_ > 0.5, the shape of the vesicle changes back from oblate to prolate in rocket orientation with its major axis perpendicular to the membrane; see Figure 1e,f.

**Figure 2:**
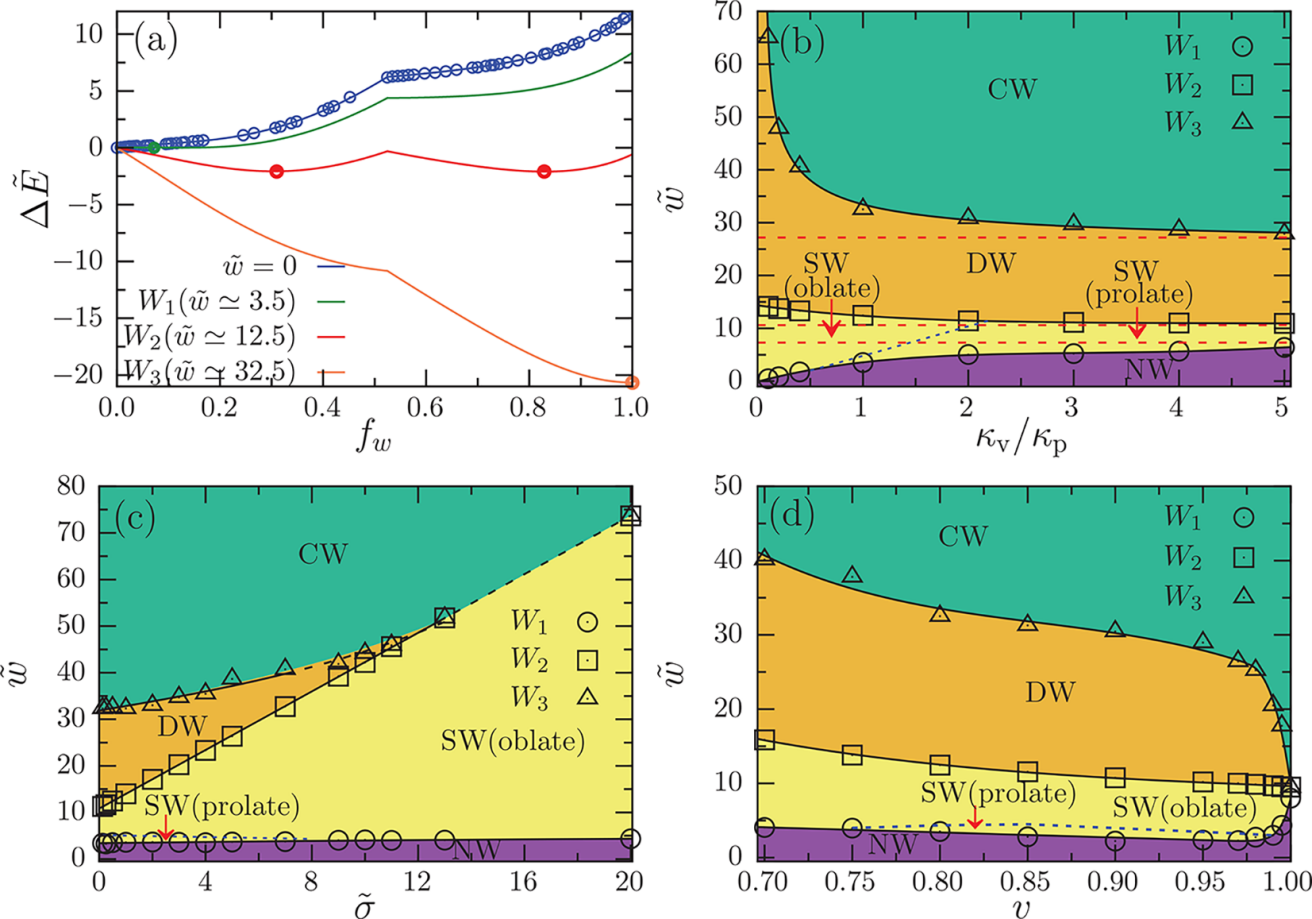
Wrapping of a nonspherical vesicle at a planar membrane. (a) Reduced wrapping energy  as a function of the wrapping fraction *f*_w_ for vesicles with reduced volume *v* = 0.8, bending rigidity ratio κ_v_/κ_p_ = 5, and reduced membrane tension . The blue circles show the numerical data for the wrapping energy in the absence of adhesion strength , and the blue line represents the fit function. The green, red, and orange lines correspond to the adhesion strengths  associated with the wrapping transitions from nonwrapped (NW) to shallow-wrapped (SW), shallow-wrapped to deep-wrapped (DW), and deep-wrapped to complete-wrapped (CW), respectively. The energies for the (coexisting) stable states at the transitions are indicated by circles. (b) Wrapping diagram in the  plane for *v* = 0.8 and . The red-dashed lines indicate the transitions for a nondeformable vesicle (κ_v_/κ_p_ → ∞) with reduced volume *v* = 0.8. (c) Wrapping diagram in the  plane for *v* = 0.8 and κ_v_/κ_p_ = 1. (d) Wrapping diagram in the  plane for κ_v_/κ_p_ = 1 and . All transitions and the stable wrapping states of the vesicles are labeled in the figures. The blue dotted lines of the prolate-to-oblate transition are guides to the eye.

In the absence of adhesion energy , a monotonic increase of the wrapping energy  is observed with increasing wrapping fraction *f*_w_; see Figure 2a. At finite adhesion strengths, depending on the elastic properties, three or four transitions between nonwrapped (*f*_w_ = 0), partial-wrapped, and complete-wrapped (*f*_w_ = 1) states are found. For adhesion strengths below the binding transition, , the nonwrapped state is stable. The binding transition *W*_1_ at adhesion strength *w*_1_ is discontinuous (see Figure S6), corresponding to the merging of two separate adhesion patches (see Figure 1). A further increase of the adhesion strength yields the coexistence of a shallow-wrapped state with low wrapping fraction (0.07 < *f*_w_ < 0.31) and a deep-wrapped state of a prolate in rocket orientation with high wrapping fraction (0.82 < *f*_w_ < 1); this *W*_2_ transition at adhesion strength *w*_2_ is also discontinuous. Within the shallow-wrapped state , a discontinuous transition from prolate in submarine orientation to oblate is observed; see Figures 2 and S6. The envelopment transition *W*_3_ at adhesion strength *w*_3_, between a deep-wrapped and the complete-wrapped state, is continuous for small and discontinuous for high tension of the planar membrane. For adhesion strengths above the envelopment transition, , the complete-wrapped state is stable. Note that wrapping transitions between partial-wrapped states are defined by barriers in the energy landscape. For practical purposes, we refer to states with wrapping fractions *f*_w_ < 0.5 as shallow-wrapped and with *f*_w_ > 0.5 as deep-wrapped.

The wrapping diagrams of the vesicle-membrane systems are obtained by systematic variation of ratio κ_v_/κ_p_ of the bending rigidities of the vesicle and the planar membrane, the reduced tension  of the planar membrane, and the reduced volume *v* that controls the shape of free vesicles; see Figure S1. For each parameter set, we compute the deformation energy  as a function of the wrapping fraction *f*_w_; see Figures S3–S5. Figure 2b shows the wrapping diagram in the  plane for fixed *v* = 0.8 and . As we decrease the value of κ_v_/κ_p_, the binding transition *W*_1_ occurs at lower adhesion strength ; in contrast, the adhesion strength for the envelopment transition increases. Therefore, as the vesicle becomes softer, binding to the planar membrane becomes easier and complete wrapping more difficult compared with wrapping a nondeformable particle (κ_v_/κ_p_ → ∞) with the same reduced volume. For the *W*_2_ transition from a shallow-wrapped to a deep-wrapped state, a very weak increase of  is observed with decreasing κ_v_/κ_p_; i.e., the stability of the SW over the DW state increases. Within the SW state, softer vesicles (κ_v_/κ_p_ ≲ 1) exhibit a stable oblate shape only, whereas stiffer vesicles (κ_v_/κ_p_ ≳ 3) exhibit a stable prolate shape only. For intermediate stiffness ratios 0.5 ≲ κ_v_/κ_p_ ≲ 3, a discontinuous shape transformation of the vesicles within the shallow-wrapped state between prolate and oblate is observed.

The effect of the tension  of the planar membrane on the wrapping diagram is shown in Figure 2c. The adhesion strength *w*_1_ for the binding transition *W*_1_ increases very weakly with increasing . The adhesion strength *w*_2_ for the shallow- to deep-wrapped transition *W*_2_ increases strongly with increasing . The adhesion strength *w*_3_ for the envelopment transition *W*_3_ increases weakly for small  but the *W*_3_ transition merges with the *W*_2_ transition for ; at high membrane tension , a stable shallow-wrapped (SW) state coexists with the complete-wrapped (CW) state. Finally, the reduced volume of the vesicles is varied in the range 0.7 ≤ *v* ≤ 1, where the shape of the free vesicles is prolate or spherical; see Figures 2d and S1. The adhesion strength *w*_1_ for the binding transition *W*_1_ initially decreases and then weakly increases with decreasing *v*. Within the SW state, as we increase the adhesion strength , a shape transformation of the vesicles from prolate to oblate is observed for 0.75 ≤ *v* < 1. The adhesion strength *w*_2_ for the shallow- to deep-wrapped transition *W*_2_ weakly increases with the decrease of *v*. The adhesion strength *w*_3_ for the envelopment transition initially increases strongly and then weakly with the decrease of *v*, accompanied by a change of the shape of the free vesicles from spherical to prolate.

**Figure 3:**
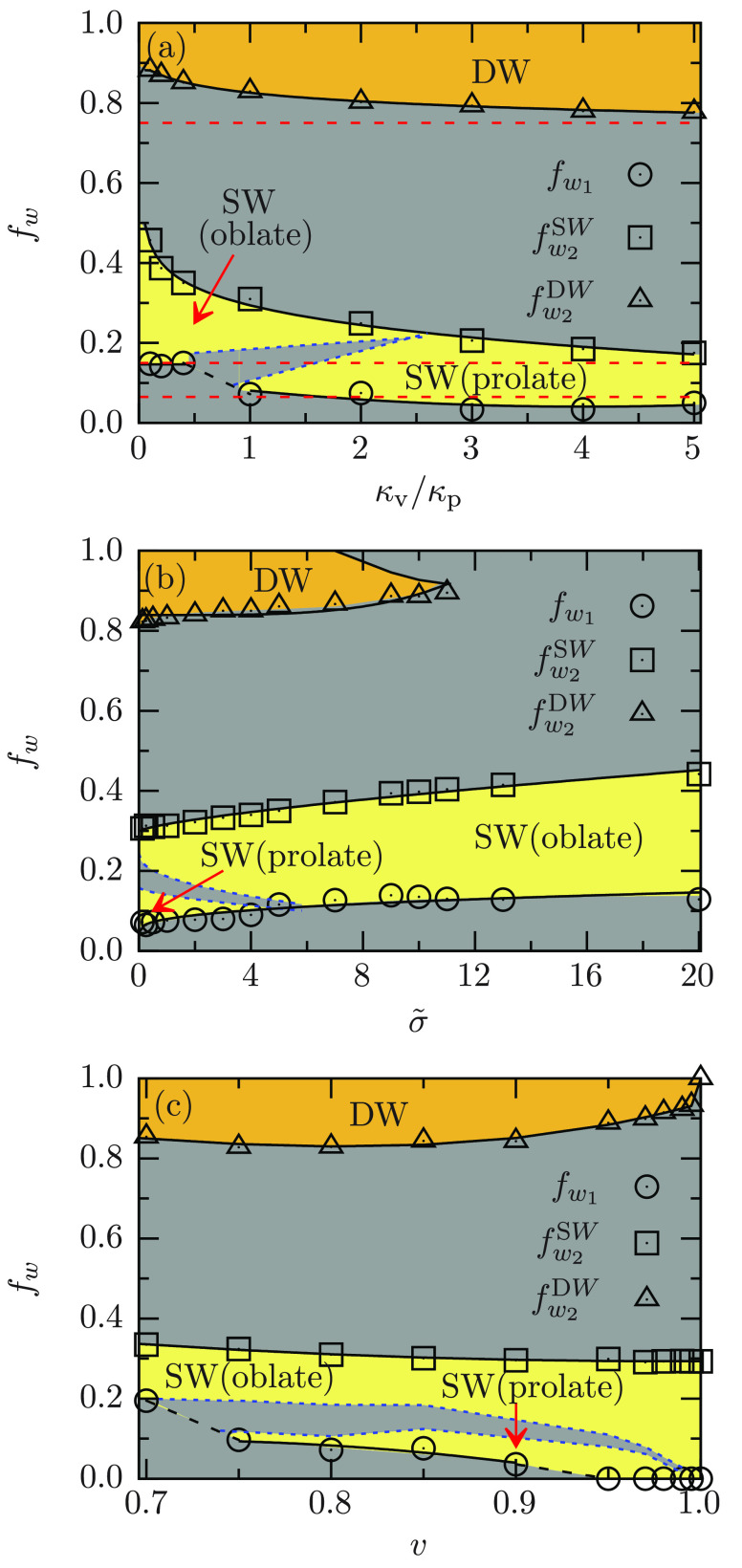
Wrapping of a nonspherical vesicle at a planar membrane. Analogous to Figure 2, the light-yellow background color indicates stable shallow-wrapped states and the dark-yellow color stable deep-wrapped states; the gray color indicates unstable and metastable states. The wrapping diagrams are presented for wrapping fractions *f*_w_ and (a) κ_v_/κ_p_ for fixed *v* = 0.8 and , (b)  for fixed *v* = 0.8 and κ_v_/κ_p_ = 1, and (c) *v* for fixed κ_v_/κ_p_ = 1 and . The black solid lines indicate *W*_1_, *W*_2_, and *W*_3_ transitions for the deformable vesicles and the red dashed lines, for nondeformable vesicles (κ_v_/κ_p_ → ∞) with reduced volume *v* = 0.8. The blue dashed lines indicate the transitions between prolate and oblate shallow-wrapped states.

Figure 3 shows the wrapping fractions for partial-wrapped states that coexist with the free vesicles at the *W*_1_ transition, for shallow- and deep-wrapped states that coexist at the *W*_2_ transition, and for deep-wrapped states that coexist with the complete-wrapped state at the *W*_3_ transition. For the *W*_2_ transition, the wrapping fractions  for the SW states and  for the DW states slowly increase with decreasing κ_v_/κ_p_ at reduced volume *v* = 0.8 and tension ; see Figure 3a. For the *W*_1_ transition, a sudden increase in wrapping fraction of the partial-wrapped state  that coexists with the nonwrapped state is observed at κ_v_/κ_p_ ≃ 1, which is associated with the shape change of the vesicle from prolate to oblate. Within the SW state, a discontinuous transition between prolate and oblate is observed in the range of bending rigidity 1 ≲ κ_v_/κ_p_ ≲ 2.5. As the vesicles become stiffer, , , and  approach the corresponding wrapping fractions for the hard particle with same reduced volume.

The wrapping fractions , , and  increase weakly with increasing tension  at reduced volume *v* = 0.8 and κ_v_/κ_p_ = 1; see Figure 3b. The coexistence of stable prolate and oblate vesicles is observed for . The envelopment transition *W*_3_ is continuous in the range . For , the envelopment transition *W*_3_ is discontinuous. For , we find a triple point where the stable SW, DW, and CW states coexist with each other. For , the DW state becomes metastable and we find coexistence of the SW and CW states.

In the range 0.7 ≤ *v* ≤ 1 for κ_v_/κ_p_ = 1 and , the wrapping fractions  and  vary weakly; see Figure 3c. For *v* = 1,  indicates that the SW state coexists with the CW state, where the envelopment transition is discontinuous. The binding transition *W*_1_ is continuous for high *v* and changes strongly between 0.7 and 0.75 for the shape change of the vesicle from oblate to prolate. For *v* ≥ 0.75,  decreases monotonically with increasing *v*, and another sudden drop is observed between *v* = 0.9 and *v* = 0.95. Such a behavior is expected as the shape of the free vesicle changes from peanut-like to ellipsoid-like. For 0.75 ≲ *v* ≲ 0.9, the shape of free vesicle is peanut-like (see Figure S1); thus, the initial attachment to the membrane leads to two patches (see Figure 1a). Therefore, the binding transition is discontinuous. The vesicle is ellipsoid-like for 0.95 ≲ *v* < 1; thus, the initial binding of the vesicle to the planar membrane occurs at the middle part and forms a single patch (see Figure S2), and the binding transition is continuous.

**Figure 4:**
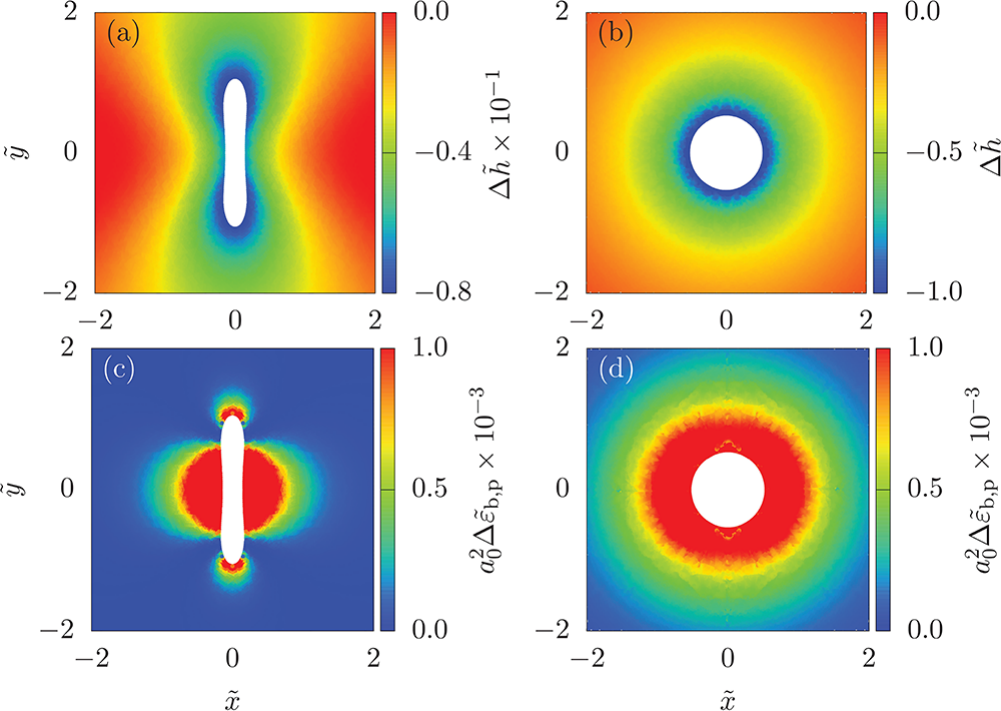
Single vesicles attached from above to initially planar membranes. The heat maps indicate (a, b) the height and (c, d) the bending energy density  of the free membrane around a vesicle with *v* = 0.8, κ_v_/κ_p_ = 5, , and  for (a, c) shallow-wrapped and (b, d) deep-wrapped states. The white areas are inside the contact line where the free membrane detaches from the vesicle.

For all partial-wrapped states, the vesicle deforms the free membrane; see Figure 4. For partial-wrapped vesicles in submarine orientation, the deviation of the free-membrane height from the plane is strongest around the tips and weakest along the sides. The local bending energy density  of the free membrane is finite at both, tips and sides, and shows lines of vanishing bending energy separating tips and sides; see Figure 4c. The free-membrane deformation at the sides can be approximated by calculations for wrapping infinitely long cylindrical particles^41,42^ and the deformation at the tips, by calculations for wrapping spherical particles.^11,43^ The free-membrane deformation along the sides raises the particle height above the optimal value for catenoid formation around the tips. The finite mean curvature along the sides and at the tips thus originate from principal curvatures with opposite signs. Therefore, the mean curvature and also the local bending energy density vanish along lines where the principal curvatures have equal magnitude. For deep-wrapped vesicles, the free membrane deformation and the bending energy distribution are cylindrically symmetric; see Figure 4d. Here, the origin of the finite bending energy of the free membrane is the membrane tension that prevents a catenoidal shape of the neck.

In summary, we have systematically studied the wrapping of single nonspherical vesicles at initially planar membranes by varying the bending rigidity ratio κ_v_/κ_p_, the reduced membrane tension , and the reduced volume *v*. The wrapping diagrams show the wrapping transitions of the vesicles from NW to SW, from SW to DW, and from DW to CW states. For deformable vesicles, the initial attachment to the membrane is easier in comparison to the hard particles. Wrapping always starts from the lowest mean curvature of the vesicles. Softer vesicles exhibit a shape change from (submarine prolate to) oblate to rocket prolate, whereas for stiffer prolate vesicles, we observe an orientation change from submarine to rocket with increasing wrapping fraction. For increasing membrane tension, we find a triple point where the SW, DW, and CW states coexist. In the limit *v* → 1 i.e., a spherical hard particle, the stable deep-wrapped state disappears.^11,43^ In general, partial-wrapped states are stabilized for deformable compared with hard particles.

### Membrane-Mediated Interactions between Two Partial-Wrapped Vesicles

The deformation of the initially planar, free membrane induces membrane-mediated interactions between two partial-wrapped vesicles. When the vesicles approach each other, both the initially planar membrane and the attached vesicles deform. We predict membrane-mediated pair interactions between shallow- and deep-wrapped prolate vesicles with *v* = 0.8 at initially planar membranes with a small finite membrane tension . The value of the adhesion strength  is chosen such that either a deep-wrapped or a shallow-wrapped state is stable. The interaction potentials are obtained by calculating the total energies of the system for various distances between the vesicles. The change in the total energy  is measured with respect to the vesicles at infinite distance, i.e., ; we calculate the total-energy difference  as a function of the reduced distance , where *d*_CL_ is the minimum contact line-to-contact line distance between the two vesicles and  is the radius of a spherical vesicle with the same membrane area. To understand the origin of the membrane-mediated interaction, we split the total potential energy  into its individual components,
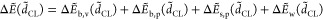
2where  is the change of bending energy of the vesicles,  is the change of bending energy of the initially planar membrane,  is the change of tension energy of the planar membrane, and  is the change of the adhesion energy.

**Figure 5:**
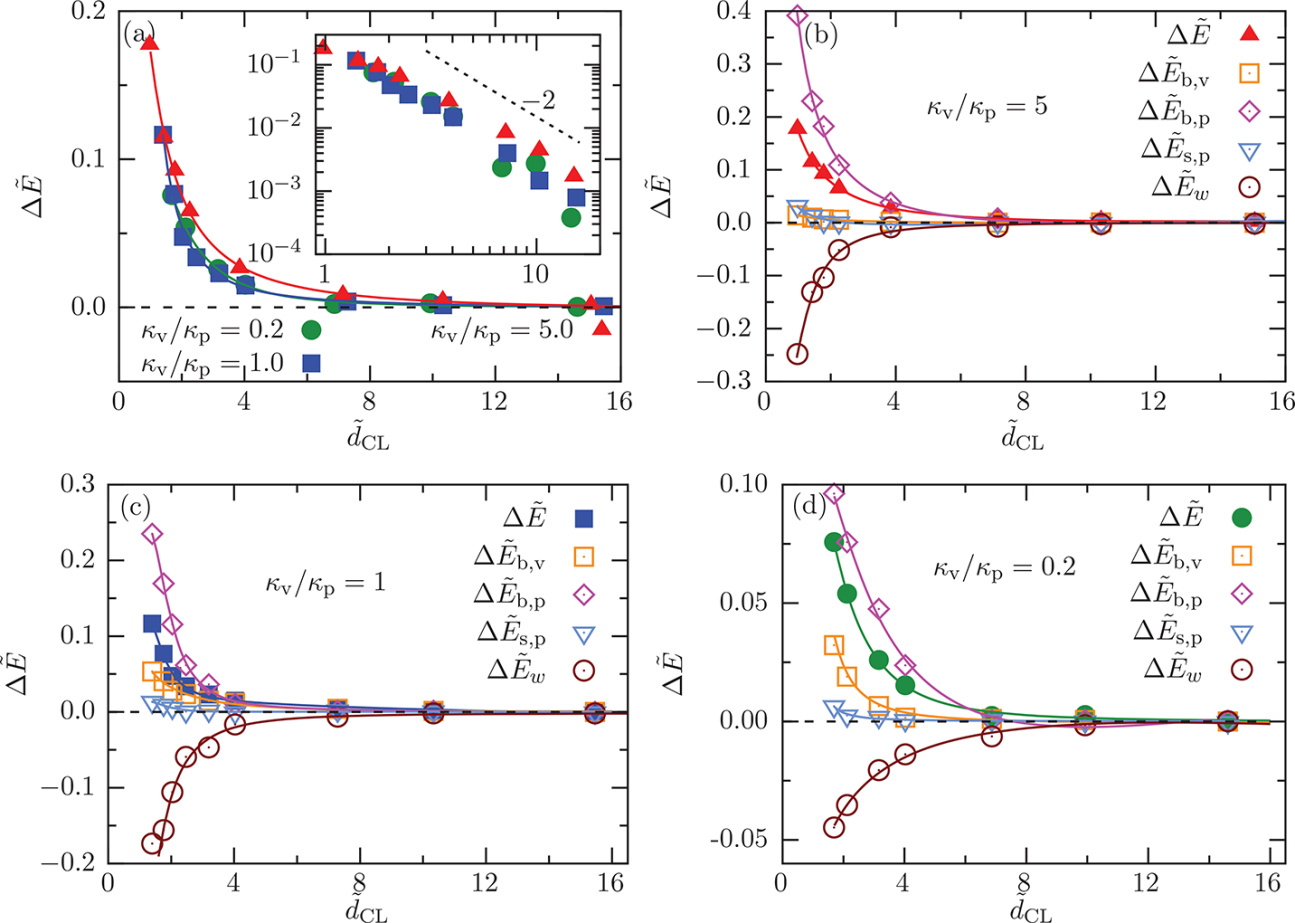
Deep-wrapped vesicles at . (a) Interaction potential  as a function of distance  for the bending rigidity ratios κ_v_/κ_p_ = 0.2, 1.0, and 5.0 at fixed *v* = 0.8, and . The inset shows the same as a log–log plot. The dashed line represents a power law with an exponent −2. The individual energy contributions: the change in bending energy of the vesicle, , the change in bending energy of the planar membrane, , the change in surface tension energy of the planar membrane,  and the change in adhesion energy, , as a function of distance  are presented for (b) κ_v_/κ_p_ = 5, (c) κ_v_/κ_p_ = 1, and (d) κ_v_/κ_p_ = 0.2.

For two deep-wrapped vesicles, , the energy  increases monotonically with decreasing distance between the two vesicles (see Figure 5a); the vesicles mutually repel each other. Within the considered distance range, the interaction potential is well described by an effective power law with exponent −2; see the inset of Figure 5a. For κ_v_/κ_p_ = 0.2, 1, and 5, we find a decreasing strength of the interaction for decreasing bending rigidity of the vesicle membrane. In Figure 5b–d, all energy components of eq 2 are plotted as a function of . The bending energy contributions,  and , both increase with decreasing distance between the vesicles. The deformation of the planar membrane increases as the vesicles become stiffer; thus, the contribution of  to the total energy increases with increasing κ_v_/κ_p_, and the relative contribution of  increases as the vesicles become softer, because the deformation of the vesicles becomes less expensive and therefore increases. The adhesion energy  becomes more negative at short distances because the adhered area increases; the change of wrapping fraction becomes less important with decreasing κ_v_/κ_p_ (see Figure S14). Thus, the contribution of  becomes weaker with decreasing κ_v_/κ_p_. We observe a weak increase of  as the distance between the vesicles decreases, which is connected to the increase of adhered membrane area as the vesicles approach each other; see Figure S14. However, the contribution of  to the total energy is negligible because a very small membrane tension is applied. For all cases, the repulsive potential mainly originates from  and .

**Figure 6:**
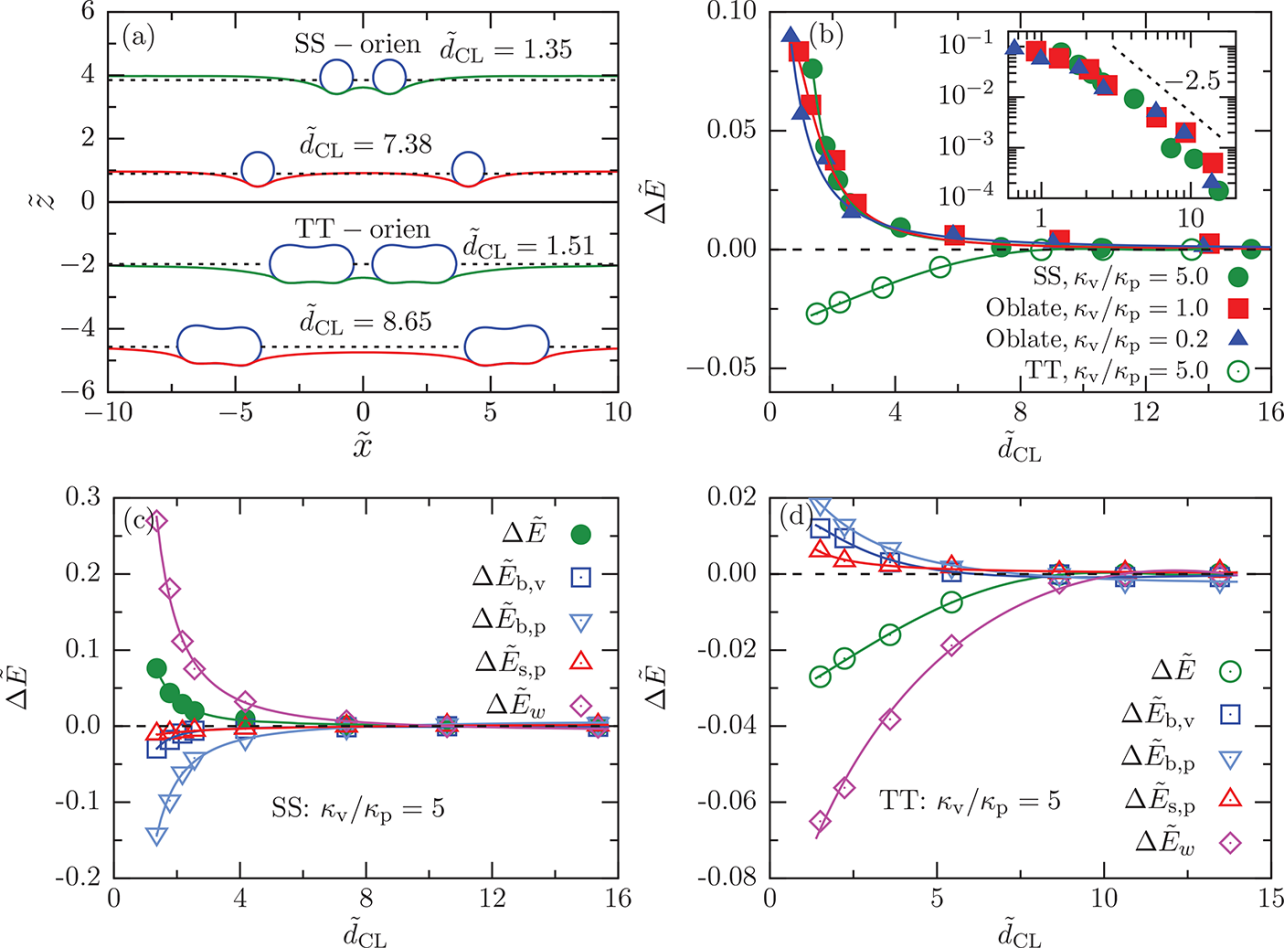
Shallow-wrapped vesicles at . (a) Cross-section side view of two vesicles with side-by-side (SS) and tip-to-tip (TT) orientations at different distances for the choice of parameters *v* = 0.8, , and κ_v_/κ_p_ = 5. (b) Interaction potential , as a function of the distance  between the two vesicles in shallow-wrapped states with SS and TT orientations. For the SS orientation, the interaction potential is plotted for the bending rigidity ratios κ_v_/κ_p_ = 0.2, 1, and 5 at *v* = 0.8 and . The inset shows the same as a log–log plot. The dashed line represents a power law with an exponent −2.5. For the TT orientation, the interaction potential is shown for κ_v_/κ_p_ = 5.0. The individual energy contributions: the change in bending energy of the vesicle, , the change in bending energy of the planar membrane, , the change in surface energy of the planar membrane, , and the change in adhesion energy, , as a function of distance  are presented at κ_v_/κ_p_ = 5 and  for (c) SS orientation and (d) TT orientation.

We also investigate the membrane-mediated interaction between two shallow-wrapped vesicles with reduced volume *v* = 0.8 and tension  for the planar membrane; see Figure 6a. The adhesion strength is fixed at , such that the shallow-wrapped state is stable for all considered values of κ_v_/κ_p_. For κ_v_/κ_p_ = 5, the interaction is repulsive for side-by-side (SS) orientation and attractive for tip-to-tip (TT) orientation; see Figure 6b. For κ_v_/κ_p_ ≤ 1, the partial-wrapped vesicles are oblate, which leads to an increase of the wrapping fraction from *f*_w_ ≈ 0.14 for κ_v_/κ_b_ = 5 to *f*_w_ ≈ 0.27 for κ_v_/κ_b_ = 1. The strength of the repulsive interaction decreases as the vesicles become softer, as for deep-wrapped vesicles.

For the SS orientation (κ_v_/κ_p_ = 5), the high curvature of the free membrane between the particles increases the bending energy cost for the free membrane; see Figure S11. A smaller area of the vesicles is adhered to the planar membrane as they approach each other, opposite to deep-wrapped vesicles where the wrapping fraction increases; see Figures 6c and S14. As a result, a qualitative change is observed in the behavior of the individual components in comparison with deep-wrapped vesicles. We find both bending energy contributions,  and , to become more negative with decreasing vesicle–vesicle distance . Also the contribution of  to the total energy decreases, which is expected because the total membrane area decreases as two vesicles approach each other. However, the contribution of  to the total energy is negligible. The change of adhesion energy  is increasing, which is the dominating contribution causing the repulsion between the vesicles. The total energy as a function of the distance can be described by an effective power law ; see the inset of Figure 6b. Our shallow-wrapped vesicles in SS orientation experience stiffer membrane-mediated interaction potentials than our deep-wrapped vesicles.

**Figure 7:**
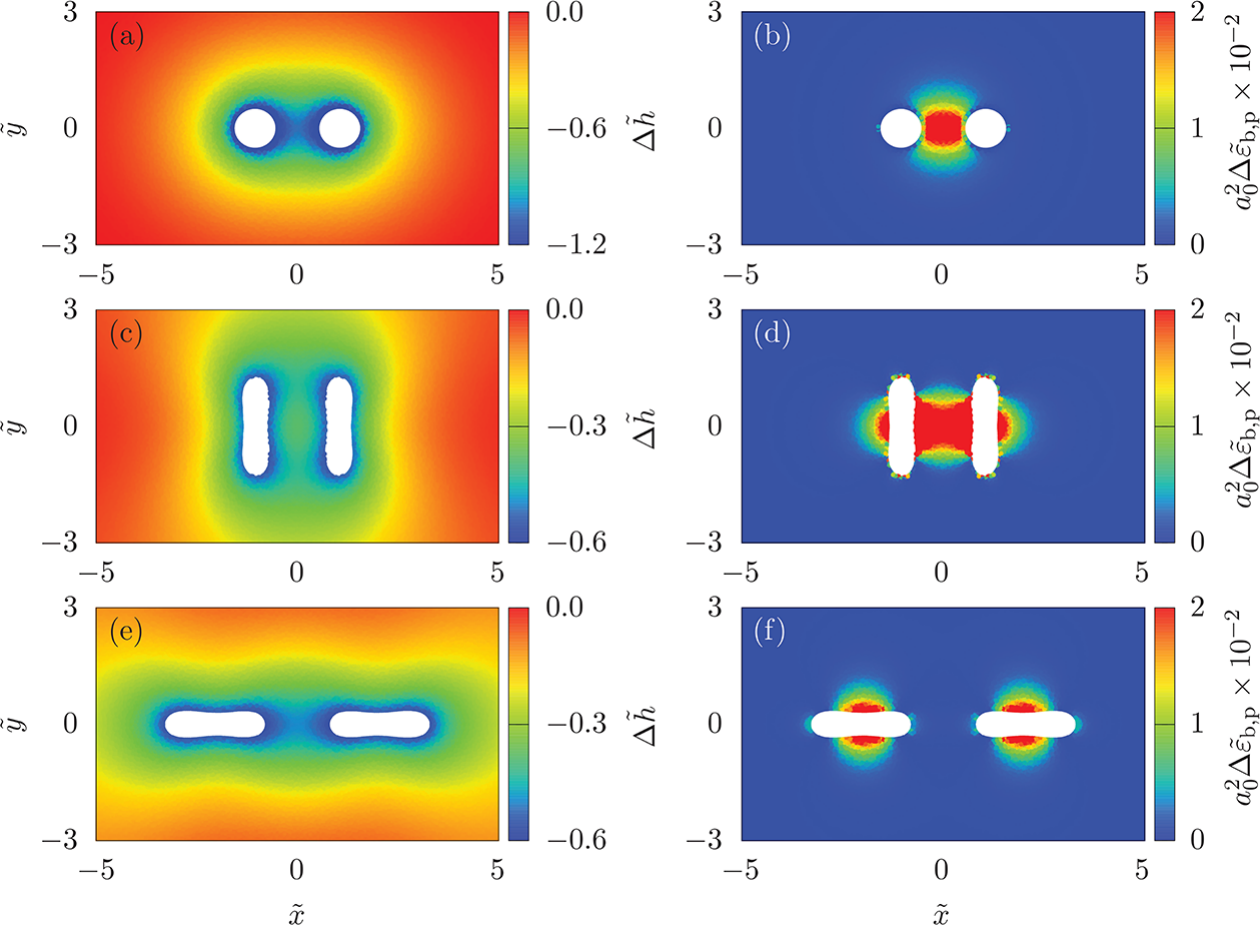
Free-membrane deformation for two vesicles at a planar membrane. (a, c, e) Local membrane height difference  with the local membrane height *h* and the height *h*_0_ of the wire frame spanning the membrane patch and (b, d, f) local bending energy density  of the free membrane. The values are shown in the *x*–*y* plane for vesicles with *v* = 0.8, reduced membrane tension , and κ_v_/κ_p_ = 5. (a, b) Deep-wrapped vesicles with their centers of mass at (*x*_1_, *y*_1_) = (−*x*_2_, *y*_2_) = (0.49, 0), (c, d) shallow-wrapped in side-by-side orientation with their centers of mass at (*x*_1_, *y*_1_) = (−*x*_2_, *y*_2_) = (0.68, 0), and (e, f) shallow-wrapped in tip-to-tip orientation with their centers of mass at (*x*_1_, *y*_1_) = (−*x*_2_, *y*_2_) = (0.75, 0).

For tip-to-tip (TT) orientation (κ_v_/κ_p_ = 5), a cooperative deformation of the membrane by the two vesicles leads to the formation of a joint “trough” for both particles without a strong increase of the deformation-energy cost of the free membrane; see Figures 6a and 7e,f. The interaction is weakly attractive. To understand this behavior, we again look into the individual energy components; see Figure 6d. In contrast to the SS orientation, here, we find that the wrapping fraction of the vesicles increases as the vesicles approach each other. As a result, tension and bending energy contributions for both the planar membrane and the vesicles increase as the distance between the vesicles decreases. The effective attraction originates from the gain in adhesion energy. For shallow-wrapped states and κ_v_/κ_p_ ≲ 1, the vesicle shape changes from prolate to oblate; therefore, there is no distinction between TT and SS orientations.

Now, we discuss shapes and local bending energies of the free membrane for both deep-wrapped and shallow-wrapped vesicles; see Figure 7. At the contact line, where the free membrane detaches from the vesicle, the membrane height deviates most from the height of the wire frame. For two deep-wrapped vesicles with contact line-to-contact line distance , the free-membrane height also strongly deviates from the wire frame height in the middle between the vesicles; see Figure 7a. Here, the deformation is strong because the membrane aims to decrease the high local bending energy cost (see Figure 7b); yet, this energy cost causes the repulsive interaction between the vesicles, compare Figure 5b. The repulsion leads to tilting of the vesicles; see Figure S8. Interestingly, the membrane-mediated interaction induces an increase of the center-of-mass height of the vesicles with respect to the wire frame; see Figure S10.

For two shallow-wrapped vesicles with contact line-to-contact line distance , the height of the free membrane deviates most strongly from the wire frame height at the tips of the vesicles; see Figure 7c. The bending energy cost of the free membrane between the vesicles is very high for the SS orientation (see Figure 7d), which leads to a partial unwrapping of the vesicle sides that face each other and therefore a repulsive interaction because of the decreasing adhesion energy when the vesicles approach each other; see Figures 6c and S12. For the TT orientation, the bending energy density of the membrane between the vesicles is very small although the membrane is also strongly deformed; see Figure 7e. The membrane height deviates the strongest from the wire-frame height at the tips that face each other, and wrapping both vesicles together increases the wrapping at the sides as well. However, the overall increase of the bending energy in the TT orientation is much lower than in the SS orientation; the joint deformation of the membrane by both vesicles strongly increases the wrapping at their sides and thereby the adhesion energy *E*_w_ (see Figures 7f and 6d). Also for shallow-wrapped vesicles, the heights of the centers of mass of the vesicles decrease with decreasing distance; see Figure S10b.

For κ_v_/κ_p_ = 0.2, we calculate the interaction potential  as a function of  for both SW and DW vesicles at different adhesion strengths; see Figure S9. For the DW state, the contribution of  and  to the total energy decreases with increasing ; thus, the interaction becomes weaker with increasing . For SW states, the deformation of the planar membrane and contribution of  to the total energy both increase with increasing . Thus, the interaction becomes stronger with increasing . However, within the considered range of adhesion strength , the interactions remain repulsive and only a quantitative change is observed in the data.

In summary, we have characterized the membrane-mediated interactions between two elastic particles. Although the interaction can be described by effective power laws, the origin is complex because it depends on the deformation energies of the initially planar membrane and the vesicle, as well as on the adhesion energy. The change of the adhesion energy with the distance between the vesicles is the largest energy component in all cases discussed above. The membrane-mediated interaction is attractive between two shallow-wrapped prolates in tip-to-tip orientation and repulsive between shallow-wrapped prolates in side-by-side orientation and between deep-wrapped prolates.

## Conclusions

In this work, we have predicted the wrapping behavior of and the membrane-mediated interactions between prolate vesicles at planar membranes. For single-vesicle systems, we systematically varied the reduced volume *v* of the vesicles, the bending rigidity ratio κ_v_/κ_p_, and the membrane tension  of the planar membrane. Wrapping always starts from the minimum curvature regions of the vesicles. Thus, initial wrapping of prolate vesicles for low adhesion strengths occurs in submarine orientation where the major axis of the vesicle is parallel to the planar membrane. With increasing adhesion strength, for stiff vesicles (κ_v_/κ_p_ > 2), the orientation changes from submarine to rocket, where the major axis of the vesicle is perpendicular to the planar membrane. For soft vesicles (κ_v_/κ_p_ < 2), a shape and orientation change of partial-wrapped vesicles from prolate submarine to oblate to prolate rocket can be observed. The binding transition, the shallow-to-deep-wrapped transition, and the envelopment transition are characterized by systematically varying the adhesion strength. The shallow-to-deep-wrapped transition is always discontinuous, whereas the binding and the envelopment transitions can be continuous or discontinuous. For fixed *v* and  the binding transition shifts to lower adhesion strengths and the envelopment transition shifts to higher adhesion strengths with decreasing bending-rigidity ratio κ_v_/κ_p_. The softer the vesicle, the easier it attaches to the membrane, but the more difficult it gets completely wrapped, which is a generic behavior of deformable particles. For fixed *v* and κ_v_/κ_p_, the binding transition is increasing weakly with membrane tension. An increase of membrane tension stabilizes shallow-wrapped states over deep-wrapped and complete-wrapped states. At very high membrane tension, the deep-wrapped state vanishes and the partial-wrapped state coexists with the complete-wrapped state; the envelopment transition is discontinuous. An interesting outlook for the single-vesicle systems is the prediction of the effect of particle softness on membrane scission. However, such calculations require local and time-dependent stresses that go beyond our equilibrium approach.^44^

The membrane-mediated interaction between two shallow-wrapped or two deep-wrapped prolate vesicles (*v* = 0.8) at planar membranes is studied by systematically varying the distance between two partial-wrapped vesicles. Here, particle systems are more complex than spherical-cap inclusions,^32,45−48^ curved membrane domains,^49^ and curved proteins^50^ for which the wrapping fraction does not depend on the distance. For deep-wrapped states of our prolate vesicles, we found the interaction potential to be repulsive until touching, which originates from the deformations of both the free membrane and the vesicles. The strength of the repulsive interaction decreases with decreasing κ_v_/κ_p_, i.e., with increasing softness of the vesicles. In the case of very deformable vesicles, attraction and clustering can be expected for distances below touching,^38^ where the conformation of the two vesicles can form a combined, near-spherical state.^51^ For shallow-wrapped states, a qualitative change in the interaction potential from attractive in tip-to-tip orientation to repulsive in side-by-side orientation is observed. Although the interaction is on the order of thermal energies, even for comparatively small membrane bending rigidities of , the deformation-mediated repulsion dominates a fluctuation-mediated Casimir attraction.^48,52,53^ For side-by-side orientation, the interaction potential is purely repulsive and becomes weaker as the vesicles become softer, which is associated with the shape change of the vesicles from prolate to oblate. Although the interaction for both, shallow-wrapped vesicles in side-by-side orientation and deep-wrapped vesicles, is repulsive, the reason is different in both cases. For side-by-side orientation, the repulsive interaction originates solely from the loss of adhesion energy as the two vesicles approach each other, whereas for deep-wrapped vesicles also the deformation interaction is repulsive. For tip-to-tip orientation, the interaction energy is attractive, which originates from the gain in adhesion energy as the vesicles approach each other.

Our theoretical predictions for wrapping and for pair interactions of soft particles at membranes can be used to optimize the shapes and the elastic properties of deformable particles for efficient use in nanomedicine, such as for targeted drug delivery. In the future, it will be interesting to investigate membrane-mediated interactions between many particles, which will help us to understand more complex problems like aggregation of virions on lipid-bilayer membranes. The understanding of the wrapping behavior of the vesicle-membrane systems is a good starting point to investigate the interaction of polymeric particles, e.g., star polymers, polymer-grafted nanoparticles, and microgels, with lipid bilayer membranes. However, contrary to vesicles, polymeric particles have bulk and shear elasticities, and their surface area increases upon binding to a membrane.^54^ Yet, the knowledge that we gained from nonspherical vesicle-membrane systems provides the basis for the estimation of membrane-mediated interactions between anisotropic elastic particles in general.

## Model and Methods

We use a continuum model for the lipid-bilayer membranes, which is applicable to systems with particle sizes that are at least a few times larger than the thickness of lipid-bilayer membranes; bending and tension energy both contribute to the wrapping process. The total energy of the system in eq 1 can be expressed by dimensionless quantities by dividing by the wrapping energy of spherical particles, πκ_p_, and expressing all areas in terms of the membrane area *A*_v_ of the vesicle. This gives
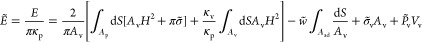
3where  and  are the reduced surface tension and reduced adhesion strength, respectively. The tension  and the pressure  serve as Lagrange multipliers, which control the reduced volume *v*. The upper bound *v* = 1 corresponds to a spherical vesicle, while *v* < 1 represents nonspherical vesicles; see Figure S1. The vesicle area *A*_v_ and enclosed volumes *V*_v_ are fixed with the help of Lagrange multipliers  and , respectively. The integrals are discretized using triangulated membranes.^55−57^

For each triangulated-membrane calculation, *A*_v_, *V*_v_, and the adhered membrane area *A*_ad_ are fixed. The area *A*_p_ of the initially planar membrane is found by energy minimization using the freely available program package “Surface Evolver”,^58^ as appropriate for a system with controlled membrane tension. To calculate minimal-energy shapes and associated deformation energies, we refine the triangulation and minimize the energy at each refinement level in an alternating sequence. The refinement of the triangles and energy minimization continues until the desired accuracy is achieved. The energy for the CW state (*f*_w_ = 1) is obtained using a system with a separate vesicle and a planar membrane. Within the accuracy of our calculations, the energy for the separated system agrees with the energy for a connected system with a very small neck between vesicle and membrane; see Figures S3–S5.

For the triangulated-membrane calculations, we exploit mirror symmetry and calculate only a quarter or half of the actual system where possible. For the deep-wrapped states, the contact line is stabilized by forcing it to a plane with an arbitrary tilt angle. Within the shallow-wrapped states, the tip-to-tip and side-by-side orientations are obtained by initializing the systems such that they evolve into the corresponding (local) minimum of the energy landscape. Here, exploiting the symmetry of the tip-to-tip and side-by-side configurations does not permit a rotation of the vesicle. Therefore, a major shape deformation associated with an energy barrier is required for changes between tip-to-tip and side-by-side orientation, and also, the energy of the (unstable) side-by-side configuration can be obtained.

For calculating wrapping diagrams and membrane-mediated interactions, we then minimize the total energy for fixed adhesion strength *w* with respect to the adhered membrane area *A*_ad_. First, the numerical data for the deformation energies are fitted for various wrapping fractions using a piecewise function, independently for submarine and rocket orientation. In each region, we fit the energy using a fourth-order polynomial
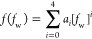
4with the coefficients *a*_0_, ..., *a*_4_. For the calculation of the phase diagrams, we then add deformation and adhesion energy, where the latter is proportional to the wrapping fraction. Binding transitions, shallow-wrapped to deep-wrapped transitions, and envelopment transitions are located by analyzing the fit functions. We take the first derivative of the total energy with respect to the wrapping fraction for different adhesion strengths. The zeros of the first derivative indicate the local minima and maxima of the wrapping energies, where the minimal represent stable or metastable states. Two minima with the same energy indicate a transition.

To calculate membrane-mediated pair interaction potentials, two vesicles are placed on the planar membrane at fixed center-of-mass distances , where . However, for the presentation of the results, we use the contact line-to-contact line distance ; we have also calculated the vesicle surface-to-surface distances  (see Table S2). For each distance, the total energy is calculated for various fixed adhered areas *A*_ad_ using Surface Evolver. To obtain the global energy minimum states, we initialized the minimization using various initial vesicle positions below the initially planar membrane and vesicle tilts and selected the lowest-energy states from several simulations for each distance before evaluating the interaction potentials. As for the single-vesicle systems, the deformation energy is fit using fourth-order polynomials. Subsequently, we minimize the energy for fixed adhesion strength *w* with respect to the adhered area *A*_ad_ for each distance. The adhesion strength  is chosen such that the minimum of  is either a stable deep-wrapped or a stable shallow-wrapped state for all vesicle–vesicle distances.

## Supporting Information Available

Shapes of nonspherical vesicles with various reduced volumes; deformation energies of single vesicles at planar membranes for various parameter sets; more detailed discussion of wrapping transitions; additional results and discussion for membrane-mediated interactions, also including membrane-mediated interactions for shallow-wrapped oblate vesicles; table for mapping center-of-mass, contact line-to-contact line, and vesicle surface–surface distances (PDF)
