# Supplementary material for: Membrane-Mediated Interactions Between Nonspherical Elastic Particles
Source: ACS Nano. 2023 Jan 20;17(3):1935–45. doi: 10.1021/acsnano.2c05801 (PMC9933614; doi:10.1021/acsnano.2c05801)
Supplement: Supplementary file 1 — nn2c05801_si_001.pdf [file nn2c05801_si_001.pdf]

Supporting Information: Membrane-Mediated Interactions Between Nonspherical Elastic Particles

Jiarul Midya,* Thorsten Auth,* and Gerhard Gompper*

Theoretical Physics of Living Matter, Institute for Biological Information Processing and Institute for Advanced Simulation, Forschungszentrum Jülich, 52425 Jülich, Germany.

E-mail: j.midya@fz-juelich.de; t.auth@fz-juelich.de; g.gompper@fz-juelich.de

Shapes of non-spherical vesicles: Non-spherical vesicles are obtained for reduced volumes $v = V_{\mathrm{v}}/V_{\mathrm{sph}} < 1$, where V_{v} is the actual volume of the vesicle and $V_{\mathrm{sph}} = (4/3)\pi a_0^3$ (where $a_0 = [A_{\mathrm{v}}/(4\pi)]^{1/2}$) is the volume of a spherical vesicle with the same membrane area, see Fig. S1. Here, we fixed the vesicle area A_{v}, and varied V_{v} to control the shape.

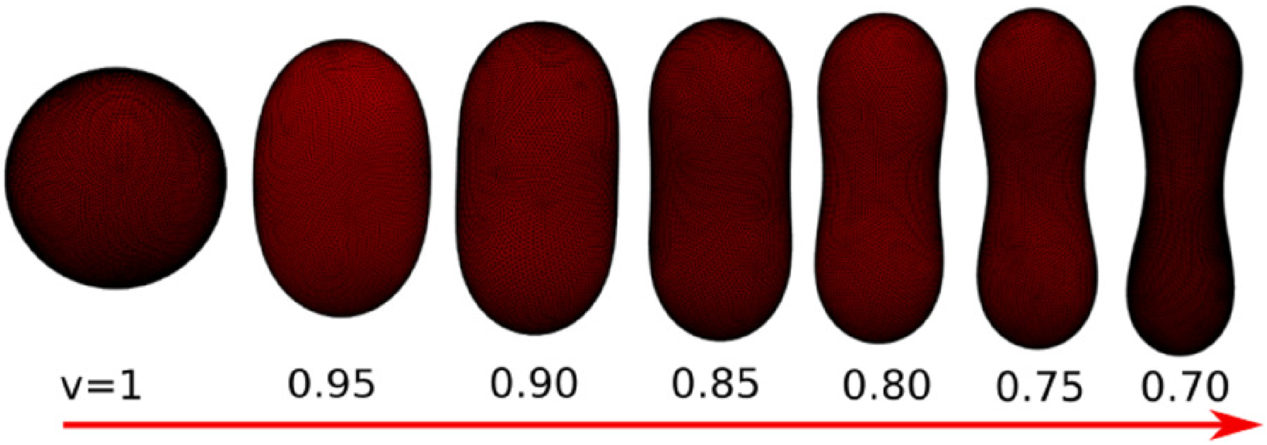

Figure S1: Shapes of vesicles with reduced volumes in the range $0.7 \leq v \leq 1$.

Wrapping of vesicles at planar membranes: Figure S2 shows wrapping of a non-spherical vesicle with $v = 0.95$ at an initially planar membrane for different wrapping fractions at fixed $\tilde{\sigma} = 0.5$ and $\kappa_{\rm v}/\kappa_{\rm p} = 1$. The wrapping starts with the lowest mean curvature regions of the vesicle membrane. Thus, initially the middle part of the vesicle gets wrapped which leads to submarine orientation where the major axis of the vesicle is parallel to the membrane surface. Here, the binding transition is continuous. As the wrapping progress, the deformation of the vesicle and the planar membrane increases with increasing wrapping fraction. For $f_w \gtrsim 0.15$, a shape transition of the vesicle is observed from a prolate to an oblate ellipsoid. The deformation of the planar membrane and vesicle are maximal at $f_w = 0.5$ when the membrane touches the rim of the oblate vesicle. For $f_w > 0.5$, the vesicle changes shape from oblate to prolate in rocket orientation, where the major axis is perpendicular to the membrane surface. Finally, a continuous wrapping transition from deep-wrapped to complete-wrapped is observed.

Calculation of deformation energies: The deformation energy of the vesicle-membrane system is calculated using curvature elasticity as given by the Helfrich Hamiltonian, see Eq. (1) in the main text. The integrals over the membrane areas are calculated numerically using triangulated membranes. Each membrane conformation is described by a set of vertices, edges, and facets. All systems are initiated with few, large facets. The triangulation is refined and the energy is minimized in an iterative procedure, assuring that neighboring triangles have similar sizes, until the triangles are sufficiently small, such that the desired accuracy is achieved.

The deformation energy increases monotonically as function of the wrapping fraction f_w, which indicates that the stable state of the vesicles is non-wrapped in absence of adhesion energy, see Figs. S3-S5. We find that the deformation energy is well described by a piece-wise function that consists of two pieces, one for low wrapping fractions and one for high wrapping fractions. Within each piece, the slope of $\Delta\tilde{E}(f_w)$ increases with increasing wrapping fraction. These fit functions for the bending energy are the basis for all data shown in the

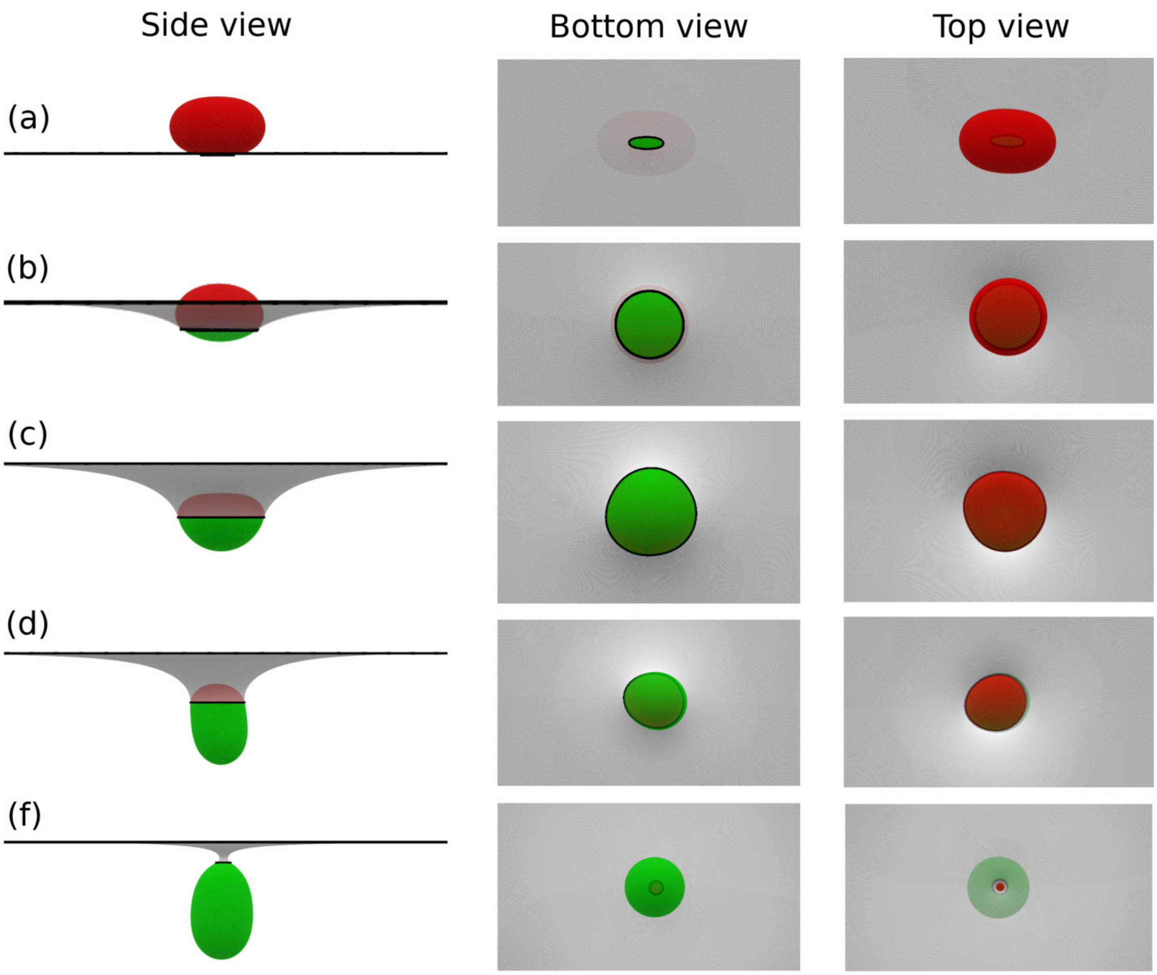

Figure S2: Wrapping of a non-spherical vesicle with $v = 0.95$ at planar membrane with reduced tension $\tilde{\sigma} = 0.5$ and bending rigidity ratio $\kappa_{\mathrm{v}}/\kappa_{\mathrm{p}} = 1$ for the wrapping fractions (a) $f_w = 0.02$ (stable state), (b) $f_w = 0.25$ (stable state), (c) $f_w = 0.5$ (unstable state), (d) $f_w = 0.75$ (stable state), and (e) $f_w = 0.98$ (stable state). The left, middle and right columns represent the side, bottom, and top views of the vesicle-membrane system, respectively.

main text. For increasing stiffness of the vesicle, the boundary between the low and the high wrapping fraction piece shifts to higher wrapping fractions, see Fig. S3. For increasing membrane tension $\tilde{\sigma}$ of the initially planar membrane, the deformation energy for fixed wrapping fraction increases, see Fig. S4. Whereas almost no effect of the tension is observed for small wrapping fractions, a pronounced effect is seen for high wrapping fractions. Finally, for prolate vesicles with various reduced volumes v, the deformation energy for high wrapping fractions increases with decreasing v, see Fig. S5.

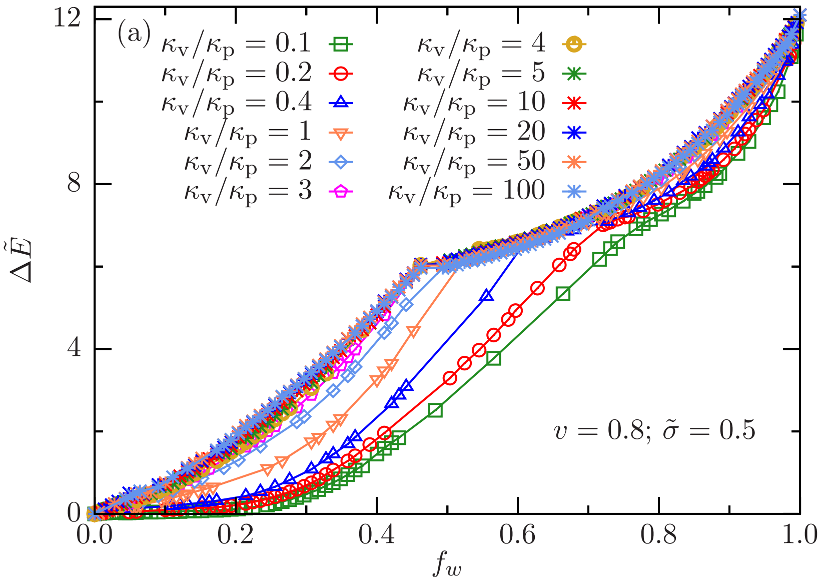

Figure S3: Deformation energy $\Delta\tilde{E}$ as function of the wrapping fraction f_w for various values of κ_v/κ_p, at fixed reduced volume $v = 0.8$ and reduced membrane tension $\tilde{\sigma} = 0.5$.

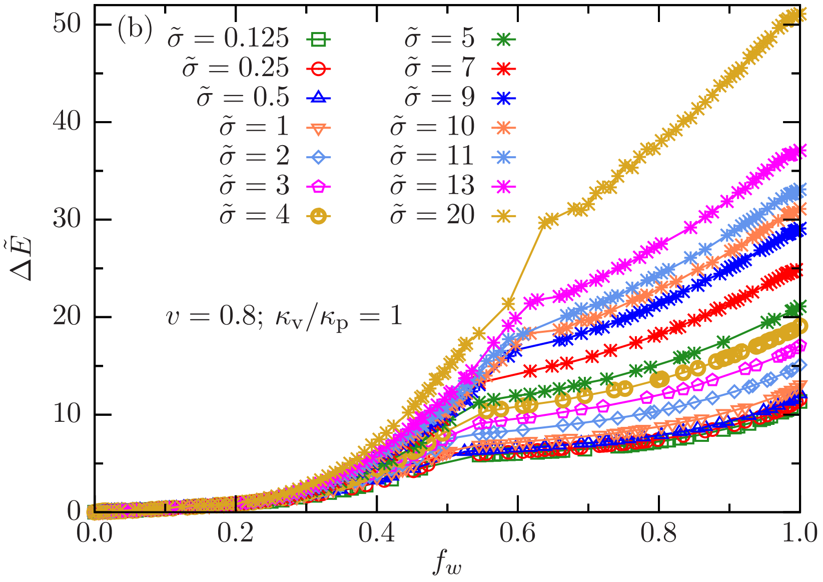

Figure S4: Deformation energy $\Delta\tilde{E}$ as function of the wrapping fraction f_w for various values of $\tilde{\sigma}$, at fixed reduced volume $v = 0.8$ and bending rigidity ratio $\kappa_v/\kappa_p = 1$.

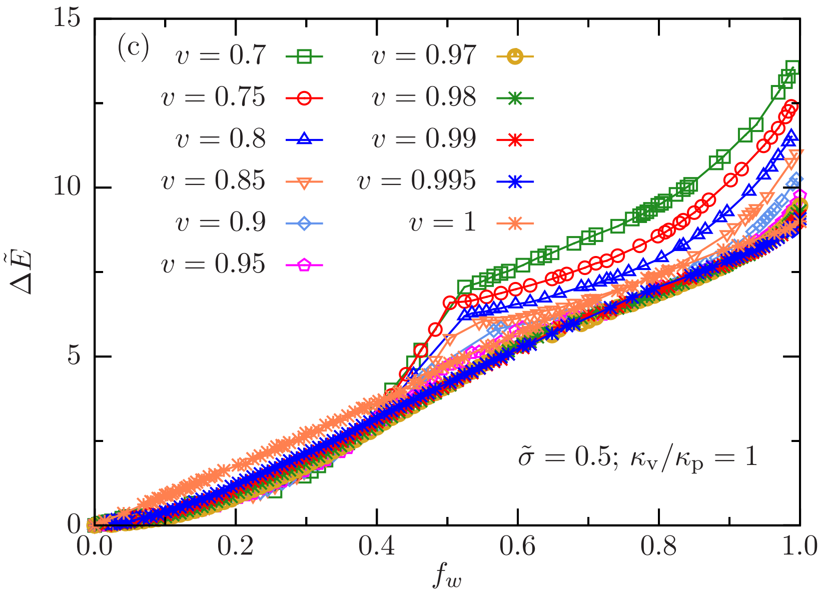

Figure S5: Deformation energy $\Delta\tilde{E}$ as function of the wrapping fraction f_w for various values of v, at fixed reduced volume $\tilde{\sigma} = 0.5$ and bending rigidity ratio $\kappa_v/\kappa_p = 1$.

Wrapping transitions: Upon increasing adhesion strength, the vesicle transitions from non-wrapped to complete-wrapped states via shallow-wrapped and deep-wrapped states. For adhesion strength $\tilde{w}_1$, a shallow-wrapped state coexists with the non-wrapped state ($f_w = 0$). In most cases, the binding transition W_1 is discontinuous as the partial-wrapped and non-wrapped states are separated by an energy barrier, see Fig. S6(a). Within the shallow-wrapped regime, a discontinuous shape transition from prolate to oblate may occur, see Fig. S6(b). Although the energy barrier is seemingly very small, the actual path of vesicle conformations for the shape transition likely involves states with higher energies.[?] For $\tilde{w}_2 > \tilde{w}_1$, we observe the coexistence of a shallow-wrapped and a deep-wrapped state; the transition W_2 is also discontinuous, see Fig. S6(c). Finally, for $\tilde{w}_3$ the slope of $\Delta\tilde{E}(f_w)$ vanishes at $f_w = 1$ in the case of a continuous envelopment transition, see Fig. S6(d). The values of reduced adhesion strengths and the associated wrapping fractions for all systems and transitions that we studied are provided in Table S1.

Wrapping diagrams: The calculation of deformation energy $\Delta\tilde{E}$ as function of wrapping fraction f_w help us to extract wrapping diagrams as shown in Fig. S7 in the $\tilde{w} - \kappa_v/\kappa_p$

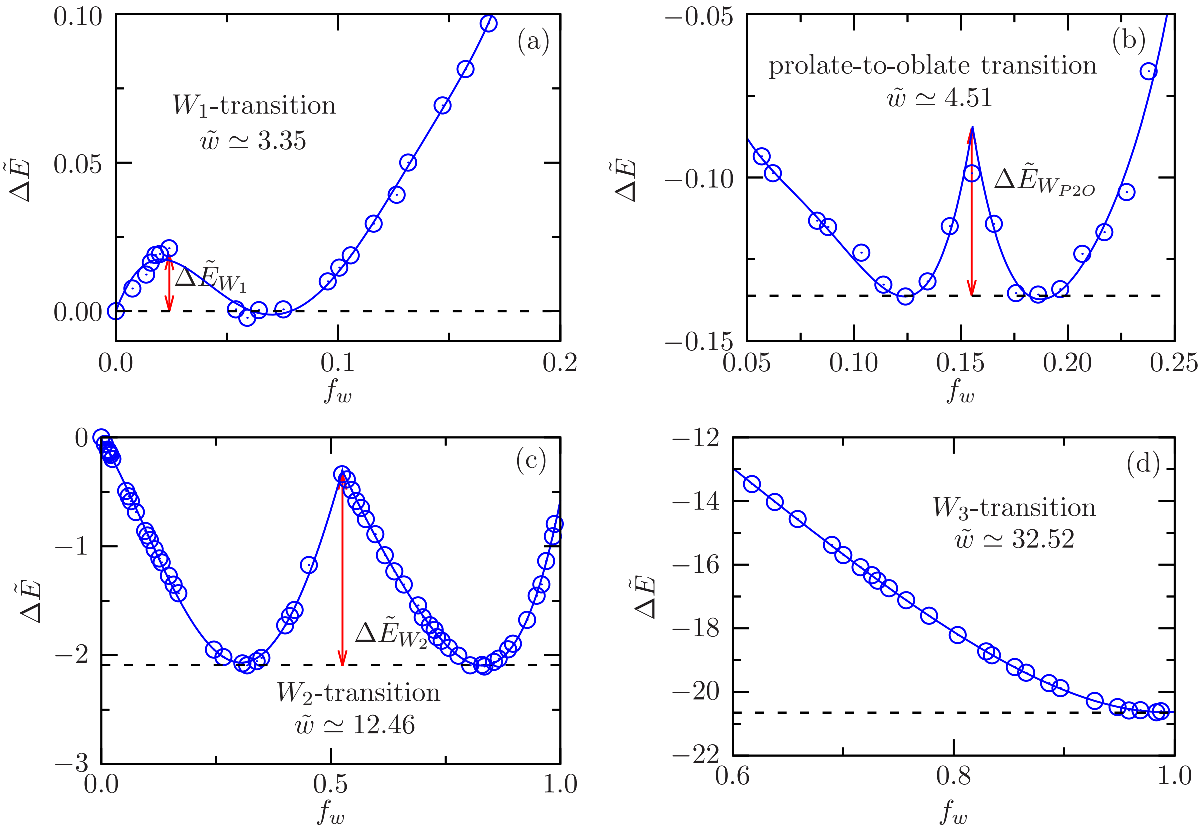

Figure S6: Total energies as function of the wrapping transitions of the vesicles at fixed $v = 0.8$, $\kappa_{\mathrm{v}}/\kappa_{\mathrm{p}} = 1$ and $\tilde{\sigma} = 0.5$. (a) Non-wrapped to partial-wrapped transition, binding transition (W_1), (b) Prolate-to-oblate transition, (c) Shallow-wrapped to deep-wrapped transition (W_2), and (d) Deep-wrapped to complete-wrapped transition, envelopment transition (W_3). The red arrows indicate energy barriers.

plane for $v = 0.8$ and $\tilde{\sigma} = 0.5$. Here, the stiffness of the vesicle is varied in the range $0.1 \lesssim \kappa_{\mathrm{v}}/\kappa_{\mathrm{p}} \lesssim 100$. We determine the adhesion strengths $\tilde{w}$ for the binding transition W_1, the shallow- to deep-wrapped transition W_2, and the envelopment transition W_3. All three values do not change much for $\kappa_{\mathrm{v}}/\kappa_{\mathrm{p}} \gtrsim 5$; we extracted values of $\tilde{w}$ for a non-deformable vesicle ($\kappa_{\mathrm{v}}/\kappa_p \to \infty$) with reduced volume $v = 0.8$ by fitting the plateaus.

Two vesicles at planar membranes: In Fig. S8, we show the cross-section side view of two vesicles in deep-wrapped states at $\tilde{w} = 15$. The height deviation of the free membrane increases with decreasing distance between the vesicles. As a result, the deformation energy of the free membrane between the two vesicles increases with decreasing distance, which leads to a repulsive pair interaction. The deviation of the center-of-mass heights of the

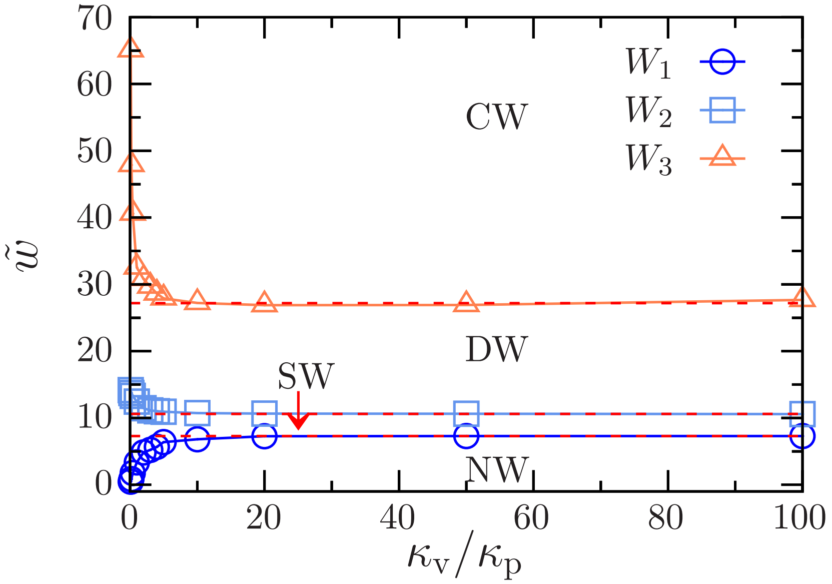

Figure S7: Wrapping diagram in the $\tilde{w} - \kappa_{\rm v}/\kappa_{\rm p}$ plan for $v = 0.8$ and $\tilde{\sigma} = 0.5$. The red-dashed lines indicate the saturation value of $\tilde{w}$ for the corresponding transitions: the adhesion strength $\tilde{w}$ for a non-deformable vesicle with $v = 0.8$.

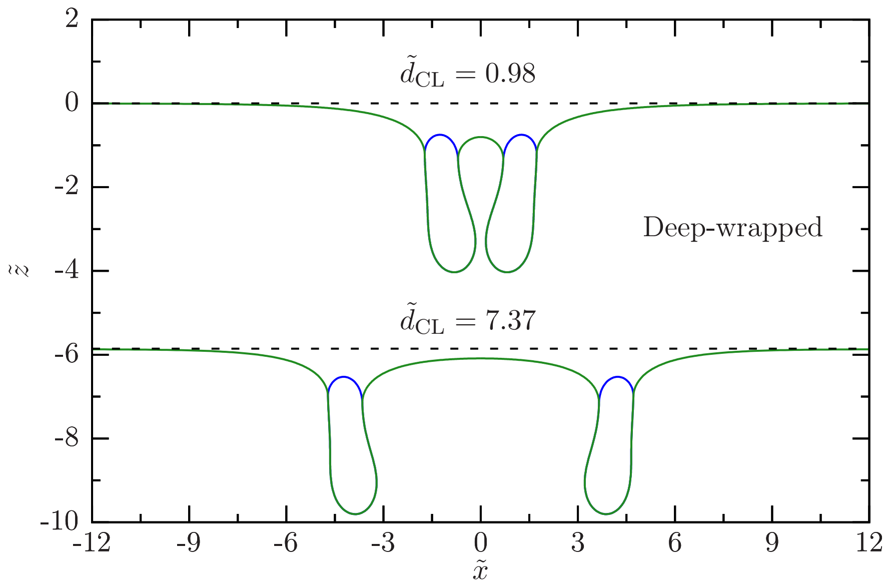

Figure S8: Cross-section side view of the two vesicles in deep-wrapped states at two different distances between the contact lines, as indicated. The parameters are $\kappa_{\rm v}/\kappa_{\rm p} = 1$, $\tilde{\sigma} = 0.5$, $v = 0.8$, and $\tilde{w} = 15$. The black dashed line indicates the height of the initially planar membrane above its wire-frame boundary.

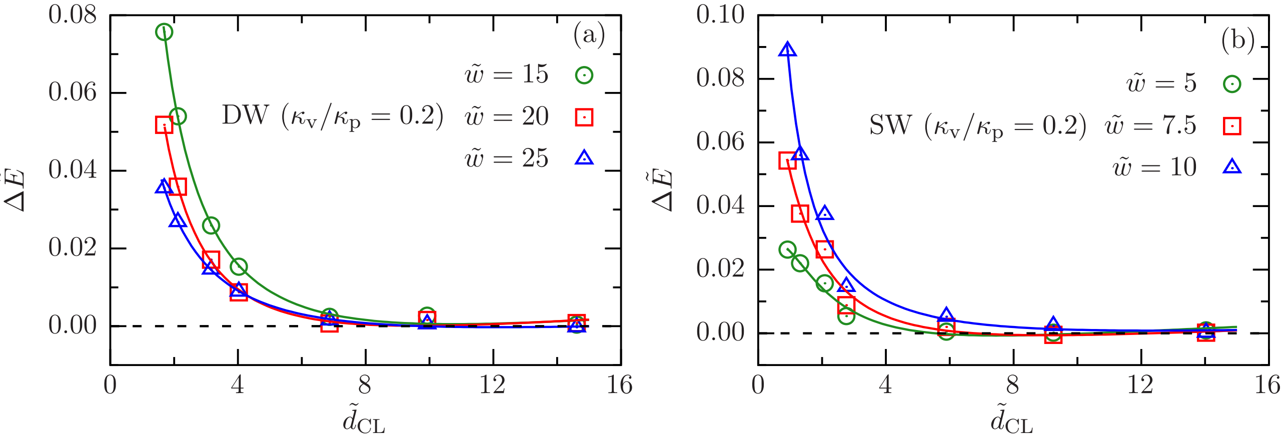

Figure S9: Membrane-mediated interaction between two vesicles with $v = 0.8$, $\tilde{\sigma} = 0.5$, and $\kappa_v/\kappa_p = 0.2$. (a) Interaction potential $\Delta\tilde{E}$ as a function of distance $\tilde{d}_{CL}$ for deep-wrapped vesicles at various adhesion strengths. (b) Same as (a) for shallow-wrapped vesicles with oblate shapes.

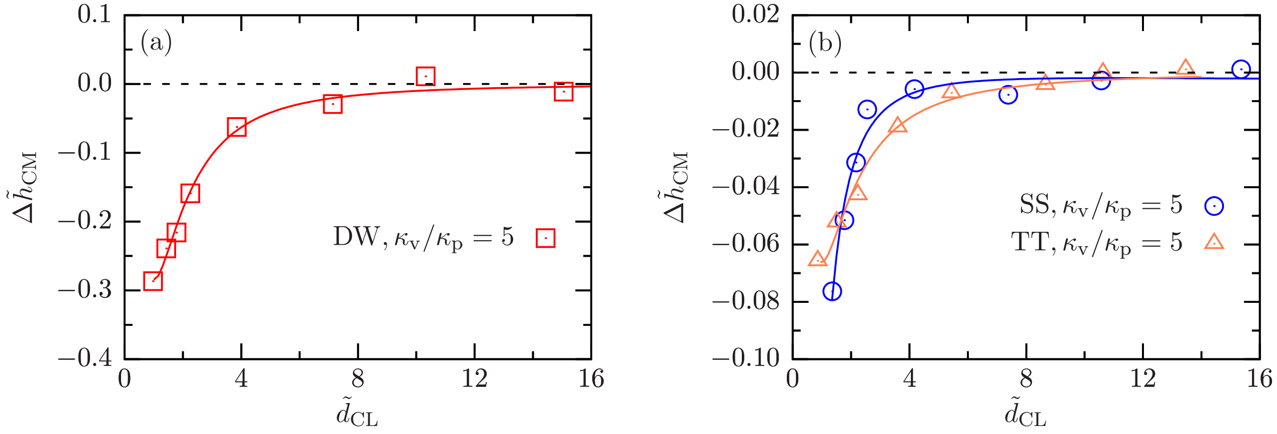

Figure S10: Relative height of the center-of-mass of the vesicles, $\Delta\tilde{h}_{CM} = \tilde{h}_{CM}(\tilde{d}_{CL}) - \tilde{h}_{CM}(\infty)$, where $\tilde{h}_{CM}(\tilde{d}_{CL})$ and $\tilde{h}_{CM}(\infty)$ are the center-of-mass height of the vesicles at distance $\tilde{d}_{CL}$ and $\tilde{d}_{CL} = \infty$, respectively, as function of distance $\tilde{d}_{CL}$. Vesicles with (a) deep-wrapped states at $\tilde{w} = 15$, and (b) shallow-wrapped states with side-by-side and tip-to-tip orientation at $\tilde{w} = 10$. The parameters are $\kappa_v/\kappa_p = 5$, $\tilde{\sigma} = 0.5$, and $v = 0.8$.

vesicles $\tilde{h}_{\rm CM}$ as function of distance $\tilde{d}_{\rm CL}$ is presented in Fig. S10. For both, deep-wrapped and shallow-wrapped states, $\Delta\tilde{h}_{\rm CM}$ becomes more negative as the two vesicles approach each other.

Figure S11(a) shows cross-section side view of two oblate vesicles with $v = 0.8$ and $\kappa_{\rm v}/\kappa_{\rm p} = 1$ on a planar membrane with tension $\tilde{\sigma} = 0.5$ at two different distances, $\tilde{d}_{\rm CL} = 2.81$ and $\tilde{d}_{\rm CL} = 8.05$. The height of the free membrane next to and, in particular, in-between the two vesicles deviates from the height of the wire frame. In-between the vesicles, the deviation increases with decreasing distance between two vesicles, see Fig. S11(b,d). The height deviation $\Delta\tilde{h}$ is maximal at the contactline, where the free membrane detaches from the vesicles. At short distance $\tilde{d}_{\rm CL} = 2.81$, the free membrane in-between the two vesicles is strongly deformed, which leads to high bending energy costs, see Fig. S11(c,e).

The various energy contributions as function of distance $\tilde{d}_{\rm CL}$ for shallow-wrapped oblate vesicles with bending rigidity values, $\kappa_{\rm v}/\kappa_{\rm p} = 1$ and $\kappa_{\rm v}/\kappa_{\rm p} = 0.2$ are presented in Fig. S12. The high curvature of the free membrane in-between the two vesicles leads to partial detachment of the vesicles. Thus, the area of the highly curved adhered membrane decreases and the bending energy of the initially planar membrane $\tilde{E}_{\rm b,p}$ decreases with decreasing distance between the vesicles. The contribution $\tilde{E}_{\rm b,v}$ to the total energy is small for small bending rigidity of the vesicle membrane. The softer vesicles adhere more to the membrane, therefore also the contribution of $\tilde{E}_{\rm s,p}$ to the total energy increases as the vesicles become softer. However, the dominating contribution comes from the adhesion energy $\tilde{E}_{\rm w}$ where the loss of adhesion energy leads to a repulsive interaction between the vesicles.

For deep-wrapped states, the relative change of bending energy of the free membrane, $\Delta\tilde{E}_{\rm b,fp}(\tilde{d}_{\rm CL}) = \Delta\tilde{E}_{\rm b,fp}(\tilde{d}_{\rm CL}) - \Delta\tilde{E}_{\rm b,fp}(\infty)$, increases with decreasing distance $\tilde{d}_{\rm CL}$ between the contact lines of the vesicles, see Fig. S13(a). Here, $\Delta\tilde{E}_{\rm b,fp}(\tilde{d}_{\rm CL})$ and $\Delta\tilde{E}_{\rm b,fp}(\infty)$ are the free-membrane bending energies at distance $\tilde{d}_{\rm CL}$ and $\tilde{d}_{\rm CL} = \infty$, respectively. The deformation energy decreases with decreasing bending rigidity of the vesicles, because for softer vesicles deforming the membrane is more expensive than deforming the vesicles. A similar behavior

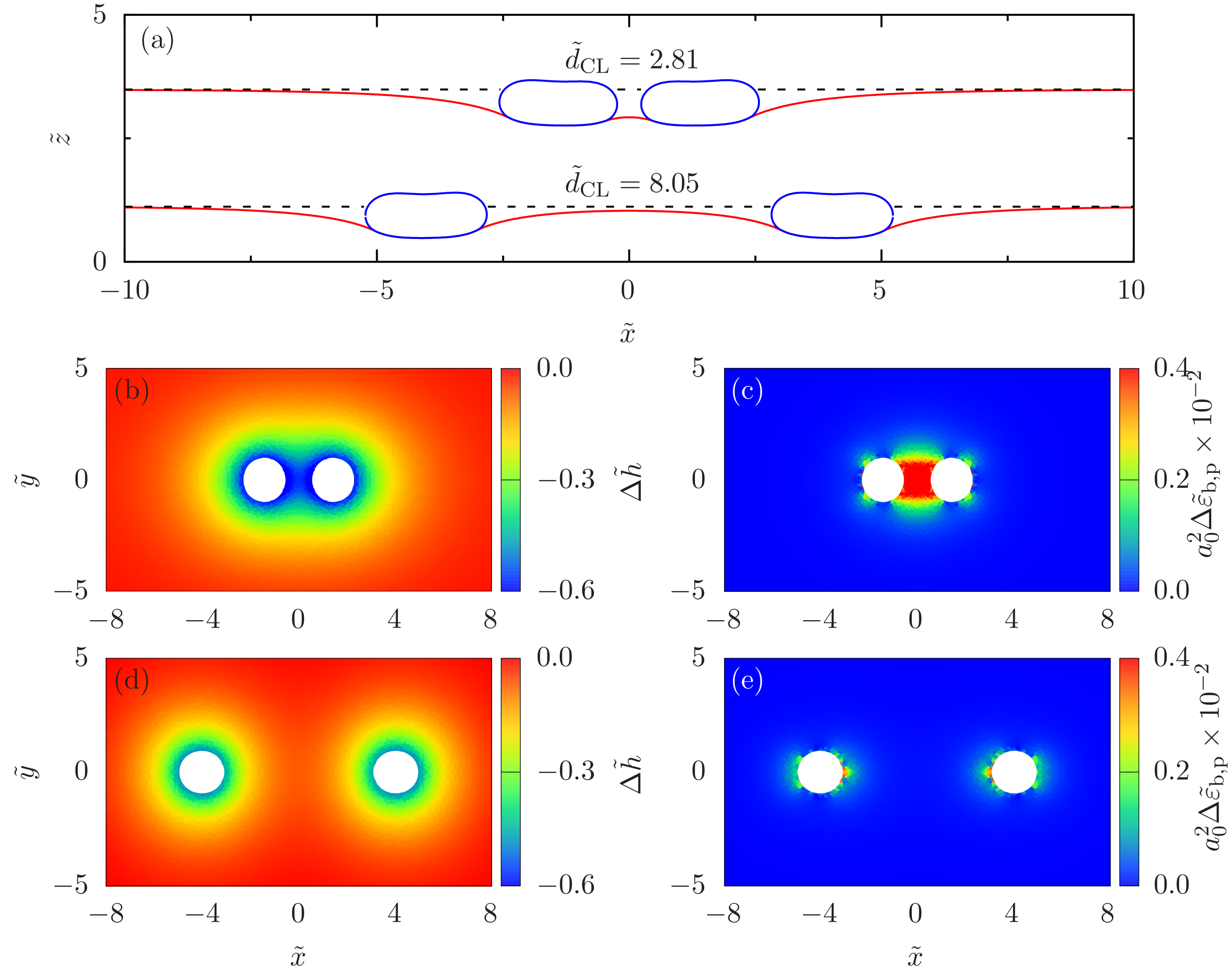

Figure S11: Shallow-wrapped oblate vesicles with $\kappa_v/\kappa_p = 1$, $\tilde{\sigma} = 0.5$, $v = 0.8$, and $\tilde{w} = 10$. (a) Cross-section side view of the two vesicles at the distances mentioned next to the vesicles. The black-dashed lines indicate the heights of the wire-frame boundary for the initially planar membrane. Free-membrane (b,d) heights and (c,e) local bending-energy densities $\Delta\tilde{\varepsilon}_{b,p}$ at distances (b,c) $\tilde{d}_{CL} = 2.81$ and (d,e) $\tilde{d}_{CL} = 8.05$. The white areas are inside the contactline where free membrane detaches from the vesicle.

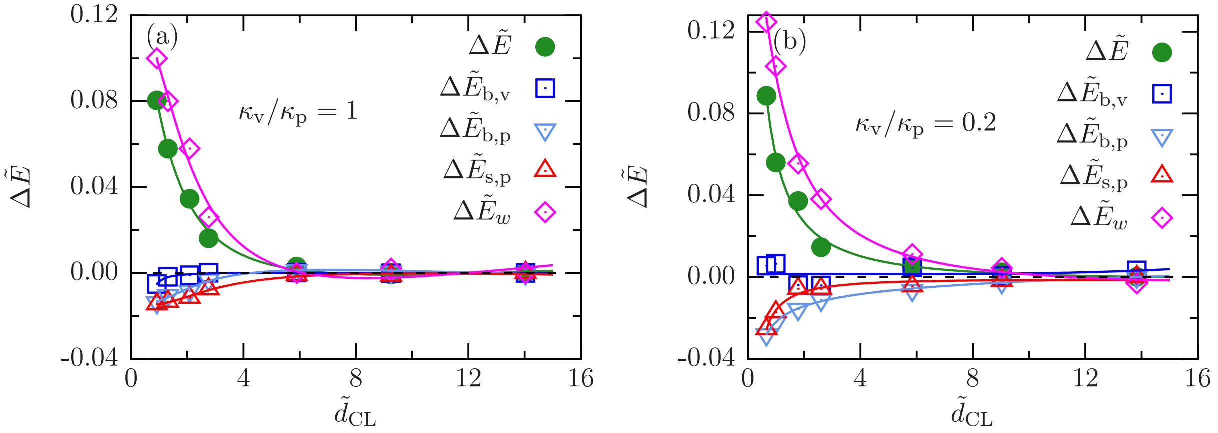

Figure S12: Shallow-wrapped oblate vesicles with $\tilde{\sigma} = 0.5$, $v = 0.8$, and $\tilde{w} = 10$. Individual energy contributions: the change in bending energy of the vesicle, $\Delta\tilde{E}_{b,v}$, the change in bending energy of the planar membrane, $\Delta\tilde{E}_{b,p}$, the change in surface energy of the planar membrane, $\Delta\tilde{E}_{s,p}$, and the change in adhesion energy, $\Delta\tilde{E}_w$, as function of distance $\tilde{d}_{CL}$ are presented for (a) $\kappa_v/\kappa_p = 1$, and (b) $\kappa_v/\kappa_p = 0.2$.

is observed for the free-membrane bending energy $\Delta\tilde{E}_{b,fp}$ of shallow-wrapped states of the vesicles, which increases as the vesicles approach each other. For $\kappa_v/\kappa_p = 5$, the increase is stronger for SS orientation of the vesicles than for TT orientation.

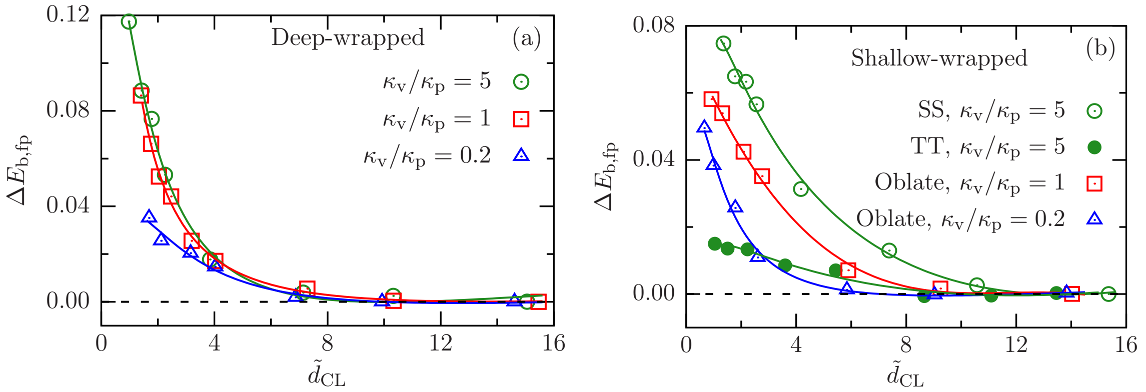

Figure S13: Relative change of bending energy of the free membrane, $\Delta\tilde{E}_{b,fp}$, as function of distance $\tilde{d}_{CL}$ for (a) deep-wrapped states at $\tilde{w} = 15$, and (b) shallow-wrapped states at $\tilde{w} = 10$ for different bending rigidity ratio, as indicated. The reduced volume of the vesicles and reduced membrane tension are fixed at $v = 0.8$ and $\tilde{\sigma} = 0.5$, respectively.

In Fig. S14, we show the relative change of wrapping fraction $\Delta f_w(\tilde{d}_{CL}) = f_w(\tilde{d}_{CL}) - f_w(\infty)$ as function of the distance $\tilde{d}_{CL}$. Here, $f_w(\tilde{d}_{CL})$ and $f_w(\infty)$ are the wrapping fractions at distance $\tilde{d}_{CL}$ and $\tilde{d}_{CL} = \infty$, respectively. For deep-wrapped vesicles, Δf_w increases

with decreasing distance $\tilde{d}_{\mathrm{CL}}$, this change becomes more prominent with increasing bending rigidity of the vesicles. A similar behavior is observed for shallow-wrapped vesicles with TT orientation, where Δf_w increases with decreasing distance between the vesicles. However, for shallow-wrapped vesicles with SS orientation and oblate vesicles, the decrease of the wrapping fraction at short distances is stronger, which indicates the detachment of the vesicles from the membrane with decreasing distance.

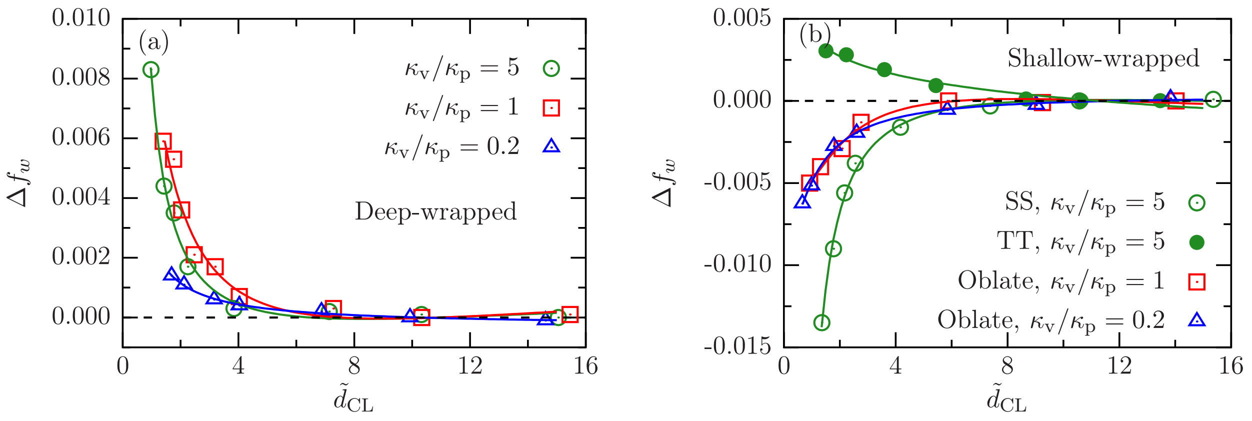

Figure S14: Relative change of the wrapping fractions of the vesicles with $v = 0.8$ and reduced membrane tension $\tilde{\sigma} = 0.5$ for different values of bending rigidity ratio κ_v/κ_p, as indicated, for (a) deep-wrapped states at $\tilde{w} = 15$, and (b) shallow-wrapped states at $\tilde{w} = 10$.

Table S1: The various columns show parameters considered for single-vesicle wrapping at planar membranes. Reduced volume v, reduced membrane tension $\tilde{\sigma}$ and bending rigidity ratio κ_v/κ_p of the vesicle and the planar membrane. Reduced adhesion strength at the binding transition $\tilde{w}[W_1]$, the shallow-wrapped to deep-wrapped transition $\tilde{w}[W_2]$, and the envelopment transition $\tilde{w}[W_3]$. Wrapping fractions at binding transition $f_w[W_1]$. The wrapping fractions of the two coexisting states, the shallow-wrapped $f_w^{\mathrm{SW}}[W_2]$ and the deep-wrapped $f_w^{\mathrm{DW}}[W_2]$, correspond to same energy at shallow-to-deep-wrapped transition.

v	$\tilde{\sigma}$	κ_v/κ_p	$\tilde{w}[W_1]$	$\tilde{w}[W_2]$	$\tilde{w}[W_3]$	$f_w[W_1]$	$f_w^{\mathrm{SW}}[W_2]$	$f_w^{\mathrm{DW}}[W_2]$
0.8	0.5	0.1	0.46	14.18	65.05	0.148	0.457	0.882
0.8	0.5	0.2	0.86	13.74	47.88	0.142	0.387	0.869
0.8	0.5	0.4	1.75	13.31	40.60	0.150	0.350	0.851
0.8	0.5	1	3.52	12.46	32.52	0.072	0.310	0.829
0.8	0.5	2	5.04	11.43	30.82	0.080	0.249	0.802
0.8	0.5	3	5.23	11.13	29.68	0.035	0.206	0.792
0.8	0.5	4	5.68	11.00	28.63	0.035	0.185	0.780
0.8	0.5	5	6.34	10.93	27.91	0.050	0.176	0.778
0.8	0.5	10	6.81	10.74	27.21	0.052	0.178	0.759
0.8	0.5	20	7.27	10.66	27.08	0.073	0.179	0.757
0.8	0.5	50	7.28	10.62	27.51	0.065	0.162	0.752
0.8	0.5	100	7.30	10.59	27.66	0.074	0.148	0.746
0.8	0.125	1	3.41	11.27	32.32	0.072	0.306	0.823
0.8	0.25	1	3.27	11.66	32.41	0.065	0.313	0.825
0.8	0.5	1	3.52	12.46	32.52	0.071	0.310	0.829
0.8	1	1	3.61	14.01	32.31	0.076	0.313	0.833
0.8	2	1	3.65	17.11	32.99	0.078	0.332	0.840
0.8	3	1	3.60	20.27	34.71	0.080	0.334	0.850
0.8	4	1	3.52	23.33	35.48	0.091	0.341	0.851
0.8	5	1	3.67	26.35	38.65	0.116	0.353	0.861
0.8	7	1	3.74	32.74	40.74	0.127	0.372	0.867
0.8	9	1	4.02	39.27	41.86	0.139	0.394	0.887
0.8	10	1	4.11	42.20	44.17	0.135	0.398	0.886
0.8	11	1	3.99	45.61	45.87	0.130	0.404	0.891
0.8	13	1	4.06	–	51.75	0.128	0.416	–
0.8	20	1	4.36	–	73.67	0.129	0.442	–
0.7	0.5	1	4.06	15.87	40.13	0.195	0.335	0.854
0.75	0.5	1	3.98	13.82	37.80	0.097	0.324	0.828
0.8	0.5	1	3.52	12.46	32.52	0.074	0.310	0.829
0.85	0.5	1	2.81	11.56	31.28	0.075	0.302	0.844
0.9	0.5	1	2.23	10.76	30.45	0.00	0.296	0.842
0.95	0.5	1	2.36	10.20	28.96	0.00	0.292	0.876
0.97	0.5	1	2.31	10.02	26.43	0.00	0.291	0.899
0.98	0.5	1	2.75	9.88	25.23	0.00	0.293	0.915
0.99	0.5	1	3.01	9.68	20.49	0.00	0.294	0.923
0.995	0.5	1	4.36	9.55	17.75	0.00	0.293	0.933
1.0	0.5	1	8.00	–	9.50	0.00	0.325	–

Table S2: Distances between the shallow wrapped ($\tilde{w} = 10$) and deep-wrapped ($\tilde{w} = 15$) vesicles at fixed $v = 0.8$ and $\tilde{\sigma} = 0.5$: reduced center-of-mass distance $\tilde{d}_{\mathrm{CC}}$, contactline-to-contactline distance $\tilde{d}_{\mathrm{CL}}$, minimum surface-to-surface distance $\tilde{d}_{\mathrm{SS}}$.

$\kappa_{\mathrm{v}}/\kappa_{\mathrm{p}}$	$\tilde{d}_{\mathrm{CC}}$	Shallow-wrapped (SS)		Shallow-wrapped (TT)		Deep-wrapped	
		$\tilde{d}_{\mathrm{CL}}$	$\tilde{d}_{\mathrm{SS}}$	$\tilde{d}_{\mathrm{CL}}$	$\tilde{d}_{\mathrm{SS}}$	$\tilde{d}_{\mathrm{CL}}$	$\tilde{d}_{\mathrm{SS}}$
5	2.01	1.36	0.78	–	–	0.98	0.59
5	2.42	1.77	1.18	–	–	1.43	0.94
5	2.82	2.17	1.59	–	–	1.78	1.44
5	3.22	2.55	1.99	–	–	2.25	1.73
5	4.03	–	–	1.50	0.74	–	–
5	4.83	4.17	3.60	2.23	1.54	3.85	3.39
5	6.44	–	–	3.60	3.16	–	–
5	8.05	7.38	6.81	5.44	4.77	7.14	6.55
5	11.27	10.57	10.04	8.66	7.99	10.32	9.82
5	13.69	-	–	11.09	10.39	–	–
5	16.10	15.36	14.86	13.46	12.81	15.05	14.70
1	2.01	–	–			1.40	0.33
1	2.42	–	–			1.76	0.70
1	2.82	0.92	0.46	For $\kappa_{\mathrm{v}}/\kappa_{\mathrm{p}} = 1$		2.04	1.23
1	3.22	1.31	0.89	vesicles are oblates;		2.47	1.62
1	4.03	2.08	1.68	no distinction		3.20	2.59
1	4.83	2.76	2.41	between SS and TT.		4.03	3.23
1	8.05	5.90	5.52			7.28	6.55
1	11.27	9.25	8.89			10.33	9.77
1	16.10	14.03	13.69			15.46	14.75
0.2	2.82	0.66	0.37			1.69	1.33
0.2	3.22	0.99	0.74	For $\kappa_{\mathrm{v}}/\kappa_{\mathrm{p}} = 0.2$		2.12	1.75
0.2	4.03	1.79	1.55	vesicles are oblates;		3.16	2.50
0.2	4.83	2.60	2.36	no distinction		4.03	3.38
0.2	8.05	5.85	5.60	between SS and TT.		6.87	6.60
0.2	11.27	9.03	8.79			9.93	9.86
0.2	16.10	13.83	13.62			14.61	14.76
